# Solving inverse problems in physics by optimizing a discrete loss: Fast and accurate learning without neural networks

**DOI:** 10.1093/pnasnexus/pgae005

**Published:** 2024-01-11

**Authors:** Petr Karnakov, Sergey Litvinov, Petros Koumoutsakos

**Affiliations:** Computational Science and Engineering Laboratory, Harvard John A. Paulson School of Engineering and Applied Sciences, Cambridge, MA 02138, USA; Computational Science and Engineering Laboratory, Harvard John A. Paulson School of Engineering and Applied Sciences, Cambridge, MA 02138, USA; Computational Science and Engineering Laboratory, Harvard John A. Paulson School of Engineering and Applied Sciences, Cambridge, MA 02138, USA

**Keywords:** partial differential equations, inverse problems, physics-informed neural networks, computational fluid dynamics

## Abstract

In recent years, advances in computing hardware and computational methods have prompted a wealth of activities for solving inverse problems in physics. These problems are often described by systems of partial differential equations (PDEs). The advent of machine learning has reinvigorated the interest in solving inverse problems using neural networks (NNs). In these efforts, the solution of the PDEs is expressed as NNs trained through the minimization of a loss function involving the PDE. Here, we show how to accelerate this approach by five orders of magnitude by deploying, instead of NNs, conventional PDE approximations. The framework of optimizing a discrete loss (ODIL) minimizes a cost function for discrete approximations of the PDEs using gradient-based and Newton’s methods. The framework relies on grid-based discretizations of PDEs and inherits their accuracy, convergence, and conservation properties. The implementation of the method is facilitated by adopting machine-learning tools for automatic differentiation. We also propose a multigrid technique to accelerate the convergence of gradient-based optimizers. We present applications to PDE-constrained optimization, optical flow, system identification, and data assimilation. We compare ODIL with the popular method of physics-informed neural networks and show that it outperforms it by several orders of magnitude in computational speed while having better accuracy and convergence rates. We evaluate ODIL on inverse problems involving linear and nonlinear PDEs including the Navier–Stokes equations for flow reconstruction problems. ODIL bridges numerical methods and machine learning and presents a powerful tool for solving challenging, inverse problems across scientific domains.

**Significance Statement**

Inverse problems are fundamental in science and engineering, often revealing solutions unattainable through direct methods. Inspired by efforts to solve such problems using physics neural networks (PINNs), we present the framework of optimizing a discrete loss (ODIL). ODIL deploys conventional discretizations on a grid instead of neural networks. ODIL is a drop-in replacement for PINNs in many scientific and engineering problems, while being several orders of magnitude more efficient in terms of computational cost and achieving higher accuracy.

## Introduction

The numerical solution of physical models expressed as partial differential equations (PDEs) is indispensable across all areas of science, engineering, and medicine. Commonly, solutions are presented for the so-called forward problems where the PDEs are discretized on grids or particles (1–3), and the discrete problems are solved to determine quantities of interest given initial and/or boundary conditions. In recent years, the advent of data science has prompted intense interest in solving PDEs in the context of “inverse problems” where the parameters and even the structure of PDE models are inferred from data (4–7). Solving inverse problems in science and engineering entails formidable challenges as the PDEs need to be solved while observing physical and geometrical constraints as well as noisy data (8). Such problems are encountered in several fields of science and engineering, and they have been handled by methods such as PDE-constrained optimization (9), data assimilation (10), optical flow in computer vision (11), and system identification (12). As there is an ever increasing number of problems that involve noisy or missing data, there is a strong need to develop efficient methods for solving such problems and machine learning has emerged as a potent modality (7, 13, 14), albeit with certain limitations (8).

Physics-informed learning using neural networks (NNs) for partial and ordinary differential equations were introduced three decades ago, by Dissanayake and Phan-Thien (15), van Milligen et al. (16), and Lagaris et al. (17). In these works, the unknown fields were represented by NNs with weights obtained by minimizing a loss function composed by the residuals of the governing equations. Related efforts used NNs for modeling nonlinear dynamical systems (18, 19), back-tracking of an *N*-body system (20), and deep NNs for reconstructing the flow field in the near-wall region of a turbulent flow using wall only information (21). At the time of their introduction, these methods were not favored for solving forward or inverse problems, in particular due to their computational cost and the speed of available computing architectures. Twenty years later, the method was revived by Raissi et al. (22) who used modern machine-learning methods and software (such as deep neural networks, automatic differentiation, and TensorFlow) to carry out its operations. The method has been popularized by the term physics-informed neural network (PINN).

PINNs have created a lot of enthusiasm in the scientific community as they offered an alternative to solving complex physical problems using convenient representations at a significantly reduced cost of implementation. However, it is broadly accepted that PINNs cannot match conventional numerical methods for solving well-posed forward problems involving PDEs but they are positioned as a convenient tool for solving ill-posed and inverse problems and data-driven modeling (7). The proper assessment of this claim hindered by the fact that to date there is no baseline for assessing the capabilities of PINNs with respect to conventional methods for solving forward and inverse problems. Raissi et al. (22) admit that conventional numerical solvers are more efficient for forward problems, a direct comparison with such solvers reveals important drawbacks of the neural network approach (23).

The overall computational cost of PINNs originates from the cost of evaluating the solution in each collocation point. Represented by a fully connected network, the solution in each point depends on all weights of the neural network, therefore the cost of evaluating it in one point is proportional to the number of weights. In contrast to neural networks, conventional grid-based methods represent the solution with local approximations resulting in a constant cost per point. Consequently, the gradient of the residual with respect to the weights in one point is a dense vector and the Hessian of the loss function a dense matrix. This makes higher order optimization methods such as Newton’s method unsuitable for training NNs. Furthermore, the convenience of representation and ease of formalism by PINNs comes with limitations. While PINN evaluates the differential operator exactly on a set of collocation points, it makes no allowance for physically and numerically motivated adjustments that speed up convergence and ensure discrete conservation such as deferred correction, upwind schemes, and discrete conservation laws as represented, for example, in the finite volume method. Furthermore, decades of development and analysis that went into conventional solvers enables understanding, prediction, and control of their convergence and stability properties. Such information is not available with neural networks and recent works aim to remedy this situation (24, 25). Application of PINN to equations with high-order derivatives is also limited (26, 27) as the computational cost of nested automatic differentiation is exponential in the order of differentiation (28, 29), while in the case of finite differences, the cost is linear.

More recently, the architectures deployed in PINNs have undergone significant enhancements, including the introduction of positional encodings, exact enforcement of boundary conditions, incorporation of adaptive activation functions, and improving the treatment of constraints and invariances (30–34). While these techniques significantly improve the performance in certain cases, they do not guarantee a universal improvement in accuracy or training speed. When it comes to studies involving PINNs, it is generally not advisable to start with these techniques, considering the numerous hyperparameters that already require tuning. For instance, there are cases where enforcing exact boundary conditions can lead to worse performance. In such scenarios, a more effective approach may involve blending the boundary conditions into the approximation, even if it diverges from the reference solution. The same principle applies to positional encodings, which entail adding sine waves to the input. While this approach clearly boosts performance in scenarios like the wave equation with periodic boundary conditions, where the solution is a linear combination of sine waves, it may not yield the same benefits for more general conditions. Nevertheless, in our experimentation with PINNs, we applied several well-known algorithmic modifications. Our observations did not indicate any noteworthy enhancements, consistent with similar experiences by other groups (35–37). Moreover, the effects of these modifications were similar to the variations observed when changing the random seed (see SI Appendix). Our observations align with a recent paper authored by one of the co-creators of PINNs, wherein the attempt to solve the Re=2000 lid-driven cavity flow problem not only was unsuccessful but also resulted in several nonphysical, pseudo-solutions (38).

Here, we present a framework that is based on conventional numerical methods that overcomes many of the challenges encountered by PINNs. We show that this valuable approach can be accelerated by five orders of magnitude by deploying conventional numerical algorithms that are facilitated by machine-learning tools. We cast the framework as optimizing a discrete loss (ODIL) by combining discrete formulations of PDEs with modern machine-learning tools to extend their scope to ill-posed and inverse problems.

The framework of ODIL minimizes a cost function for the discrete forms of PDEs using gradient-based and Newton’s methods. We demonstrate that ODIL is effective in solving inverse problems for equations with missing parameters and with limited data. This framework has two key aspects. First, the discretization itself that defines the accuracy, stability, and consistency of the method. Second, the optimization algorithm to solve the discrete problem. Our method uses efficient computational tools that are readily available for both of these aspects. The discretization properties are inherited from the conventional numerical methods building upon the advances in this field over multiple decades. Since the sparsity of the problem is preserved, the optimization algorithm can use a Hessian and achieve a quadratic converge rate, which remains out of reach for gradient-based training of neural networks. We remark that solving the discrete equations as a minimization problem is not a new idea. It has been developed in various forms in different domains, including the discretize-then-differentiate approach in the context of PDE-constrained optimization (9), as the penalty method (39), and is related to the 4D-VAR problem in variational data assimilation (10, 40), in addition to regularization techniques (41). Differentiable solvers (42–45) serve as a tool for computing the gradients of the solution with respect to parameters of the problem. They can be combined with a gradient-based optimization method to fit the solution to known data and infer the parameters. However, they assume that the forward problem is well-posed, which is not always the case as demonstrated by the “notorious test problem” in optimal control (46, 47) discussed in Fig. S4.

Another related approach is the neural bootstrapping method (NBM) (27, 48). NBM represents the solution with a neural network as in PINN but evaluates conventional discretization schemes on a set of random collocation points using a local stencil around each point. The authors have applied this approach to elliptic problems with discontinuities. Although NBM does not reduce the cost of evaluating the solution in each point introduced by a fully connected NN, it can be less computationally expensive than PINN if the equations contain high-order derivatives. The cost of nested automatic differentiation in PINN grows exponentially with the order of the derivative, but NBM uses finite differences for which the cost is linear. While the authors do not provide a direct comparison with conventional solvers in terms of the accuracy and computational cost, they use small neural networks (e.g. 1,000 parameters) and evaluate the loss on a grid with significantly more cells (e.g. $128^{3}$ cells), suggesting that NBM is more efficient than conventional solvers in terms of the number of required degrees of freedom. However, the presented test cases involve smooth functions with only low-frequency modes (e.g. products of sinusoidals). Given the phenomenon of spectral bias (49) of NNs, more complex solutions will require larger networks. Moreover, the authors do not demonstrate applications of NBM to inverse problems, which is the primary domain of ODIL. In a related work, blended inverse-PDE networks (BiPDE) (50) incorporate a differentiable PDE solver as a layer in an NN with the goal of solving inverse problems for parameterized PDEs. BiPDE is an autoencoder that takes a solution field, passes it through an encoder to the unknown hidden parameters, and uses the parameters as input for the PDE solver layer, which outputs the solution. The autoencoder is trained on data obtained by solving the forward problem with random parameters sampled from a given distribution. The trained encoder can be used for finding the parameters from a known solution field, possibly perturbed by noise. We remark that, unlike BiPDE, ODIL includes both the residual of the discretized PDEs and known data into the loss function and obtains the solution and the parameters by minimizing the total loss.

In the following, we present a potent computational framework for solving forward and inverse problems with missing and noisy data and facilitate its deployment by adopting machine-learning tools. Adopting modern machine-learning tools, such as automatic differentiation, renders the implementation of ODIL as convenient as applying gradient-based methods in PINNs, providing a baseline for the comparison of these methods. We present a number of solutions to prototypical and benchmark problems to evaluate the performance of ODIL. Our results indicate that ODIL outperforms PINNs by up to five orders of magnitude in computational speed and with better accuracy while covering the same class of inverse and ill-posed problems. Finally, we note that ODIL is an example of bridging numerical methods and machine learning. The successful fusion of these two domains with complementary potentials can create further developments of computational methods for challenging scientific and engineering problems described by PDEs.

## Methods

### Formulation of ODIL

Here, we formulate the ODIL framework to solve forward and inverse problems for PDEs. Using a given discretization of the PDEs, we construct a loss function from the residuals of the discretization, data terms, and regularization terms. The applications in this article involve discretizations on a uniform Cartesian grid, but this general formulation is applicable to other cases, e.g. an unstructured triangular grid. Let *C* denote the cell indices of a grid in a *d*-dimensional space $R^{d}$ with the number of cells $N_{C}=|C|$. Consider a system of $N_{F}$ discrete equations, $N_{u}$ unknown fields, and $N_{\theta }$ parameters


(1) $$F^{\left(i\right)}[u,\theta ]=0,\;i=1,\ldots ,N_{F},$$

where $F^{\left(i\right)}\;:\;(R^{C}{)}^{N_{u}}\times R^{N_{\theta }}\to R^{C},\;i=1,\ldots ,N_{F}$ are the residual operators of the discrete equations, $u=(u^{\left(1\right)},\ldots ,u^{\left(N_{u}\right)})$, are the unknown discrete fields $u^{\left(j\right)}\in R^{C}$, and $\theta \in R^{N_{\theta }}$ is the vector of parameters. These discrete equations can represent discretizations of the governing PDEs (e.g. a finite difference scheme), equality constraints to impose known data (e.g. measurements of the solution in a finite set of points), and regularization terms (e.g. discrete Laplace operator as a smoothness constraint). The parameters *θ* include all unknowns that are not discrete fields (e.g. weights of neural networks used to represent nonlinear terms). The relevant boundary and initial conditions can either be incorporated into the discretization of the PDEs or added as extra equality constraints. The ODIL framework solves the unconstrained minimization problem


(2) $$\min_{u,\theta }L(u,\theta )$$

with the loss function


(3) $$L(u,\theta )=\sum_{i=1}^{N_{F}}\left[\frac{1}{N_{C}}\sum_{c\in C}(F_{c}^{\left(i\right)}[u,\theta ]{)}^{2}\right],$$

where $\cdot_{c}$ denotes the value of a field in cell c∈C.

We consider two approaches to solving this minimization problem. The first approach is to use a standard gradient-based method, such as Adam (51) or L-BFGS-B (52), with the gradients of L(u,θ) computed using automatic differentiation (53). One important enhancement of this approach is the multigrid decomposition technique to accelerate the convergence, which is described further below.

The second approach is the Gauss–Newton method, which iteratively solves the problem by updating the solution based on linearization of the residual operators about the current solution. Let $\left(u^{s},\theta^{s}\right)$ denote the solution at iteration *s*. Define the linear approximations to the operators $F^{\left(i\right)}$ about the point $\left(u^{s},\theta^{s}\right)$


(4) $$\begin{array}{rl} & {\tilde{F}}^{(i),s}[u,\theta ]\\ & \;=F^{\left(i\right)}[u^{s},\theta^{s}]+{\frac{\partial F^{\left(i\right)}}{\partial u}|}^{s}[u-u^{s}]+{\frac{\partial F^{\left(i\right)}}{\partial \theta }|}^{s}[\theta -\theta^{s}], \end{array}$$

where $\cdot {|}^{s}$ indicates that the Jacobian is evaluated at $\left(u^{s},\theta^{s}\right)$. Using the linearized operators, define the approximate loss function


(5) $$L^{s}(u,\theta )=\sum_{i=1}^{N_{F}}\left[\frac{1}{N_{C}}\sum_{c\in C}({\tilde{F}}_{c}^{(i),s}[u,\theta ]{)}^{2}\right],$$

which is now a quadratic function of u and *θ*. A minimum of this function provides the solution at the next iteration


(6) $$(u^{s+1},\theta^{s+1})=\arg\min_{\left(u,\theta \right)}L^{s}(u,\theta ).$$

On the other hand, a minimum of $L^{s}$ satisfies the optimality conditions


(7) $$\begin{array}{c} \nabla_{u}L^{s}(u^{s+1},\theta^{s+1})=0,\\ \nabla_{\theta }L^{s}(u^{s+1},\theta^{s+1})=0, \end{array}$$

which is a system of linear equations. To derive the system, we combine all unknowns in one column vector


(8) $$U=(u,\theta )\in R^{N_{u}N_{C}+N_{\theta }},$$

all residuals in one column vector


(9) $$F=(F^{\left(1\right)},\ldots ,F^{\left(N_{F}\right)})\in R^{N_{F}N_{C}},$$

and the corresponding derivatives in one matrix


(10) $$A=\left[\begin{array}{cc} \frac{\partial F^{\left(1\right)}}{\partial u} & \frac{\partial F^{\left(1\right)}}{\partial \theta }\\ \vdots & \vdots \\ \frac{\partial F^{\left(N_{F}\right)}}{\partial u} & \frac{\partial F^{\left(N_{F}\right)}}{\partial \theta } \end{array}\right]\in R^{\left(N_{F}N_{C})\times (N_{u}N_{C}+N_{\theta }\right)}.$$

In this notation, the linearized operators (4) take the form


(11) $${\tilde{F}}^{s}[U]=F^{s}+A^{s}(U-U^{s}),$$

where $F^{s}=F[U^{s}]$. The approximate loss function (5) becomes


(12) $$L^{s}(U)=\frac{1}{N_{C}}\|{\tilde{F}}^{s}[U]{\|}_{2}^{2}=\frac{1}{N_{C}}\|F^{s}+A^{s}(U-U^{s}){\|}_{2}^{2}.$$

Finally, the optimality conditions (7) are now equivalent to


(13) $$(A^{s}{)}^{*}A^{s}(U^{s+1}-U^{s})=-(A^{s}{)}^{*}F^{s}.$$

Solving this linear system for $U^{s+1}$ provides the iteration update. To solve the resulting sparse linear system at each iteration, we use a direct method (54, 55) or an algebraic multigrid method (56). Iterative methods require an initial guess to start the iteration. Unless stated otherwise, we use a zero initial guess for the discrete fields in all presented applications. We note that if the discretization is linear and the corresponding optimization problem is quadratic, the Gauss–Newton method converges to the solution after one iteration from any initial guess. If a problem involves a neural network, the initial weights are sampled from a uniform distribution and the initial biases are zero. For brevity, we refer to the above Gauss–Newton method as simply Newton’s method throughout the article.

### Calculating sparse Jacobians

Discrete equations (1) that result from approximations of PDEs are often local, i.e. the residual in each cell depends on values of unknown fields in a limited number of neighboring cells. In such cases, the Jacobians with respect to the unknown fields in Eq. (10) are sparse. To calculate them, we intend to use automatic differentiation of scalar functions. Coloring techniques (57, 58) can be used to estimate a sparse Jacobian using multiple evaluations of a Jacobian-vector product. However, we use a more explicit approach that directly tracks the dependencies on the neighboring cells. For simplicity, assume that $N_{u}=1$. Cases with $N_{u}>1$ are handled by extension. To calculate the Jacobian of a residual operator $F\;:\;R^{C}\times R^{N_{\theta }}\to R^{C}$, we assume that the operator explicitly depends on the field values in *K* neighboring cells


(14) $$F[u,\theta ]=g[S^{\left(1\right)}[u],\ldots ,S^{\left(K\right)}[u],\theta ],$$

where $g\;:\;(R^{C}{)}^{K}\times R^{N_{\theta }}\to R^{C}$ is an element-wise operator of its field arguments, i.e. its value $g_{c}[v^{\left(1\right)},\ldots ,v^{\left(K\right)},\theta ]$ in each cell c∈C only depends on $\left(v_{c}^{\left(1\right)},\ldots ,v_{c}^{\left(K\right)},\theta \right)$. Here $S^{\left(k\right)}\;:\;R^{C}\to R^{C},\;k=1,\ldots ,K$ are shift operators selecting values from the neighboring cells or possibly the identity operator. On a Cartesian grid, a discrete field is a *d*-dimensional array and a shift operator simply shifts the array. On an unstructured triangular grid, a shift operator selects adjacent triangles. Then, we introduce a scalar function


(15) $$G(v^{\left(1\right)},\ldots ,v^{\left(K\right)},\theta )=\sum_{c\in C}g_{c}(v^{\left(1\right)},\ldots ,v^{\left(K\right)},\theta )$$

and calculate the desired Jacobian as


(16) $$\frac{\partial F}{\partial u}=\sum_{k=1}^{K}\frac{\partial G}{\partial v^{\left(k\right)}}⊙S^{\left(k\right)},$$

where the derivatives of G are evaluated at $v^{\left(k\right)}=S^{\left(k\right)}[u]$ and ⊙ is the element-wise product. Note that Eq. (16) expresses the Jacobian through *K* derivatives of the scalar function G as intended.

### Multigrid decomposition

Here, we propose a multigrid technique to accelerate the convergence of optimization methods for problems that involve discrete fields on a grid. Multigrid methods are generally accepted as the fastest numerical methods for solving elliptic differential equations (59). A standard multigrid method consists of the following parts: a hierarchy of grids including the original grid and coarser grids, discretizations of the problem on each grid, interpolation operators to finer levels, and restriction operators to coarser levels. The method iteratively updates the solution on each level, interpolates the update to finer levels, and restricts the residuals to coarser levels.

As discussed above, the optimization problem in ODIL can be solved with Newton’s method which involves a linear system at each step, so the multigrid method can be applied directly to that linear system. However, then we need to construct the linear system explicitly and rely on a general multigrid solver which may have limited efficiency especially on GPUs (60). Furthermore, if the equation involves unknown parameters, such as parameters of a neural network, as demonstrated on the problem of inferring conductivity from temperature, in Results, the corresponding Jacobian matrix is dense, which makes Newton’s method impractical.

Instead of Newton’s method, we can solve the problem with a general optimization algorithm, such as Adam or L-BFGS. However, as we demonstrate on the lid-driven cavity flow in Results, they require orders of magnitude more iterations than Newton’s method. To accelerate the convergence, we propose the following technique. Consider a uniform grid with $N_{1}=N$ points in each direction. Introduce a hierarchy of successively coarser grids of size $N_{i}=N/2^{i-1}$ for i=1,…,L, where *L* is the total number of levels. Define the multigrid decomposition operator as


(17) $$M_{L}[u_{1},\ldots ,u_{L}]=u_{1}+T_{1}u_{2}+\ldots +T_{1}T_{2}\cdots T_{L-1}u_{L},$$

where each $u_{i}$ is a field on grid $N_{i}$, and each $T_{i}$ is an interpolation operator from grid $N_{i+1}$ to the finer grid $N_{i}$. The multigrid decomposition of a discrete field *u* on a grid of size *N* reads


(18) $$u=M_{L}[u_{1},\ldots ,u_{L}].$$

Note that this representation is over-parameterized and therefore not unique. The total number of scalar parameters increases from $N^{d}$ of the original field *u* to $N_{1}^{d}+\cdots +N_{L}^{d}$ for the representation $u_{1},\ldots ,u_{L}$. We define the interpolation operators $T_{i}$ using linear interpolation (59). The rest of the framework remains the same, including the discretized PDEs and the optimization algorithm. Gradients of the resulting loss function can be computed using automatic differentiation. This technique addresses the issue of locality of gradient-based optimizers by extending the domain of dependence of each scalar parameter so that information can propagate through the grid faster. Figure S3 demonstrates the speedup on the lid-driven cavity flow. We refer to Ref. (61) for further details and examples related to the multigrid decomposition technique. All applications of ODIL with a gradient-based optimizer presented in Results use this technique. The only exception is the wave equation, where we intend to compare ODIL and PINN using standard optimization methods without enhancements.

## Results

### Wave equation: accuracy and cost of PINN

We apply ODIL and PINN to solve an initial-value problem for the wave equation in two dimensions and compare both methods in terms of accuracy and computational cost. The problem consists of the wave equation


(19) $$u_{tt}=u_{xx}$$

with the initial conditions and boundary conditions


(20) $$\begin{array}{c} u(x,0)=U(x,0)\\ u_{t}(x,0)=U_{t}(x,0)\\ u(-1,t)=U(-1,t)\\ u(1,t)=U(1,t) \end{array}$$

in a rectangular domain (x,t)∈Ω=[−1,1]×[0,1], where u(x,t) is the unknown function and U(x,t) is the exact solution


(21) $$U(x,t)=\frac{1}{10}\sum_{k=1}^{5}(\text{cos}(x-t+0.5)\pi k+\text{cos}(x+t+0.5)\pi k).$$

Both methods reduce the problem to minimization of a loss function. ODIL represents the solution on a uniform Cartesian grid of size $N_{x}\times N_{t}$ with cell centers $x_{i}=-1+(i+0.5)\Delta x,\;i=1,\ldots ,N_{x}$, and $t^{n}=(n+0.5)\Delta t,\;n=1,\ldots ,N_{t}$, where $\Delta x=2/N_{x}$ and $\Delta t=1/N_{t}$. A second-order finite volume discretization yields the following residual operator


(22) $$F_{i}^{n}[u]=\frac{u_{i}^{n}-2u_{i}^{n-1}+u_{i}^{n-2}}{\Delta t^{2}}-\frac{u_{i+1}^{n-1}-2u_{i}^{n-1}+u_{i-1}^{n-1}}{\Delta x^{2}}$$

for n>2 and


(23) $$\begin{array}{c} \;F_{i}^{2}[u]=\frac{u_{i}^{2}-u_{i}^{1}}{\Delta t^{2}}-\frac{U_{t}(x_{i},0)}{\Delta t}-\frac{u_{i+1}^{1}-2u_{i}^{1}+u_{i-1}^{1}}{\Delta x^{2}}\\ F_{i}^{1}[u]=U(x_{i},0)+U_{t}(x_{i},0)-u_{i}^{1} \end{array}$$

for n=1,2 derived using the initial conditions. The boundary conditions enter the discretization through values in the halo cells set to


(24) $$\begin{array}{c} \;u_{0}^{n}=\frac{1}{3}(u_{2}^{n}-6u_{1}^{n}+8U(-1,t^{n}))\\ u_{N_{x}+1}^{n}=\frac{1}{3}(u_{N_{x}-1}^{n}-6u_{N_{x}}^{n}+8U(1,t^{n})) \end{array}$$

using second-order extrapolation. ODIL solves the minimization problem for the loss function [3] computed from this residual operator. We choose $N_{x}=N_{t}$ and define the number of parameters of ODIL as $N=N_{x}N_{t}$. PINN represents the solution as a fully connected neural network and evaluates the residuals of the differential equation exactly on a finite set of collocation points (22). The network consists of two equally sized hidden layers with tanh activation functions. We define the number of parameters *N* of PINN as the total number of weights. The number of collocation points for PINN amounts to 8,192 points inside the domain and 768 points for the initial and boundary conditions. The optimization problem is solved with L-BFGS-B or Newton for ODIL and with L-BFGS-B for PINN. Here, we do not use the multigrid decomposition technique of ODIL.

**Fig. 1.**
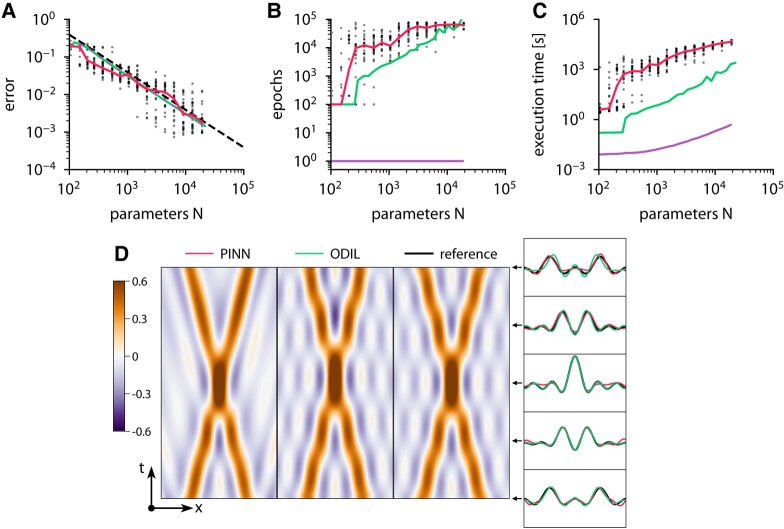
Wave equation solved using PINN and ODIL. A–C) Root-mean-square error relative to the exact reference solution (A), required number of epochs (B), and execution time on one CPU core (C) of various methods: PINN with L-BFGS-B 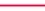, ODIL with L-BFGS-B 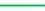, ODIL with Newton 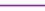, and line with slope 1/N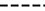. For PINN, the dots show samples with random initial weights and collocation points, and solids lines show the median value. The number of parameters is the number of weights for PINN and the number of grid cells for ODIL. D) Solution obtained using PINN with L-BFGS-B 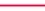 with two hidden layers of 25 neurons, ODIL with Newton on a grid of 25×25 cells 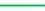, and exact reference solution 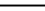.

Figure 1 presents examples of the solutions obtained using both methods and also shows how the error and execution time of both methods depend on the number of parameters. The results of PINN include 25 runs with random initial weights and positions of the collocation points. In the case of ODIL, solutions from two optimization methods, L-BFGS-B and Newton, coincide. For each run of PINN or ODIL with L-BFGS-B, we determine the best error achieved during 80,000 epochs and estimate the required number of epochs as the number of epochs to achieve 150% of the best error. Then, we estimate the execution time per epoch as the mean over the last 100 epochs for L-BFGS-B and one epoch for Newton’s method, excluding the time spent on startup, building the computational graph, and output. The measurements are performed on one CPU core of Intel Xeon E5-2690 v3 and exclude the startup time spent on building the computational graph required on the first epoch. We report the execution time as the product of the required number of epochs and the execution time per epoch. Both methods demonstrate similar accuracy. The error of ODIL scales as 1/N corresponding to a second-order accurate discretization. The error of PINN is significantly scattered, but the median error also scales as 1/N. In addition, we refer to Fig. S5 that shows the effect of weights initialization and demonstrates how PINN behaves on an asymmetric solution. The execution times of the methods, however, are drastically different. With N≈19,000 parameters, PINN with L-BFGS-B takes 13 h, ODIL with L-BFGS-B takes 35 min, and ODIL with Newton takes only 0.5 s, which is about 100,000 times faster than PINN. We note that running the same test on a GPU would reduce the timings of PINN since it creates a significant workload for a GPU. However, the timings obtained here on one CPU core serve as an estimate of the overall computational cost of each method, i.e. the number of required floating point operations.

### Poisson equation in two dimensions

We apply ODIL and PINN to solve the boundary-value problem for the Poisson equation


(25) $$u_{xx}+u_{yy}=f(x,y)$$

with zero Dirichlet boundary conditions in a unit domain $(x,y)\in [0,1{]}^{2}$. The exact reference solution is an oscillatory function


(26) $$u(x,y)=\text{sin}(\pi (kx{)}^{2})\text{sin}\;\pi y$$

with k=2 or k=4 and the right-hand side f(x,y) is found by substituting the exact solution into the equation, i.e. using the method of manufactured solutions. ODIL solves the problem on a grid of 64×64 cells. PINN uses a neural network of size 32×32×32 with 400 points on the boundaries and 1,000 collocation points inside the domain, which are resampled every 100 iterations. Both methods use the Adam optimizer with the learning rate set to 0.001 for PINN and 0.005 for ODIL. To implement the PINN method, we use the DeepXDE framework (62).

**Fig. 2.**
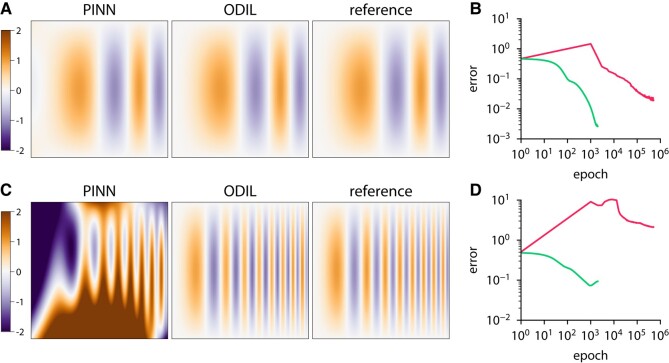
Poisson equation solved using PINN and ODIL. The exact reference solution is an oscillatory function $u(x,y)=\text{sin}(\pi (kx{)}^{2})\text{sin}\pi y$. A, B) Solution and history of root-mean-square error obtained using PINN with Adam 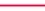 and ODIL with Adam 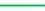 compared to the exact reference solution for k=2. C, D) Same for k=4.

Figure 2 shows results of both methods compared to the reference solution along with the convergence history. The error is measured relative to the exact solution. In the case k=2, both methods recover the reference solution achieving an error of 0.02 for PINN and 0.002 for ODIL. However, PINN takes about 100 times more iterations to achieve the same error. In the case k=4, PINN fails to recover the reference solution after 500,000 epochs, while ODIL achieves an error of 0.1.

### Velocity from tracer

**Fig. 3.**
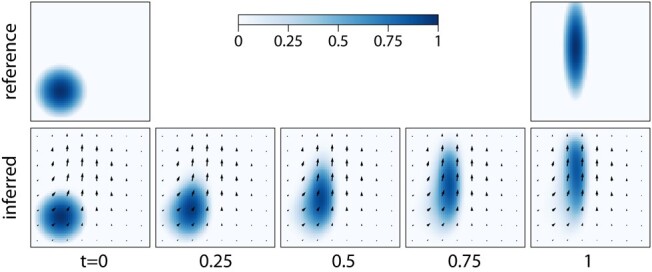
Inferring the velocity field from two snapshots of a tracer. The tracer satisfies the advection equation with an unknown velocity, and the reference tracer is imposed at the first and final time instants. Arrows show the inferred velocity field.

We consider the evolution of a tracer field governed by the advection equation in two dimensions. The problem is to find a velocity field u(x,y) given that the tracer field c(x,y,t) satisfies the advection equation


(27) $$\frac{\partial c}{\partial t}+u\cdot \nabla c=0$$

in a unit domain, and the initial $c(x,y,0)=c_{0}(x,y)$ and final $c(x,y,1)=c_{1}(x,y)$ profiles are known. The discrete problem is solved in space and time on a 64×64×64 grid. The loss function includes a discretization of the equation with a first-order upwind scheme, terms to impose known initial and final profiles of the tracer, and regularization terms $\|10^{-3}\nabla^{2}u{\|}^{2}$ and $\|u_{t}{\|}^{2}$ to prioritize velocity fields that are smooth and stationary. The results of inference are shown in Fig. 3. The inferred velocity field stretches the initial tracer representing a circle to match the final profile.

Inferring the velocity field from tracers is an example of the optical flow problem (11) in computer vision and the image registration problem (63) in medical imaging. Furthermore, reconstructing the velocity field from a concentration field can assist experimental measurements.

### Inferring conductivity from temperature

Here, we consider an inverse problem of inferring a conductivity function from temperature measurements. We solve the problem in a unit domain $(x,t)\in [0,1{]}^{2}$. The problem is to find a nonlinear conductivity function k(u) and temperature field u(x,t) that satisfies the heat equation


(28) $$u_{t}-(k(u)u_{x}{)}_{x}=0$$

with zero Dirichlet boundary conditions u(0,t)=u(1,t)=0 and initial conditions u(x,0)=U(x), where U(x)=g(x)−g(0) and $g(x)=e^{-50(x-0.5{)}^{2}}$. In addition, the temperature field needs to take known values $u(x_{j},t_{j})={\tilde{U}}_{j}$ in a finite set of measurement points $\left({\tilde{x}}_{j},{\tilde{t}}_{j}\right)$ for $j=1,\ldots ,N_{\mathrm{data}}$ with $N_{\mathrm{data}}=200$. We discretize the equation on a uniform grid of $N_{x}\times N_{t}$ cells with a second-order Crank–Nicolson scheme such that the residual operator takes the form


(29) $$\begin{array}{rl} F_{i}^{n}[u,k] & =\frac{u_{i}^{n}-u_{i}^{n-1}}{\Delta t}\\ & -\frac{k(u_{i+1/2}^{n-1/2})(u_{i+1}^{n-1/2}-u_{i}^{n-1/2})-k(u_{i-1/2}^{n-1/2})(u_{i}^{n-1/2}-u_{i-1}^{n-1/2})}{\Delta x^{2}} \end{array}$$

for $i=1,\ldots ,N_{x}$ and $n=1,\ldots ,N_{t}$, where $u_{i+1/2}=(u_{i+1}+u_{i})/2$, $u^{n-1/2}=(u^{n}+u^{n-1})/2$, and k:R→R is a conductivity function. The initial and boundary conditions enter the discretization through values in the halo cells defined as


(30) $$\begin{array}{c} \;\;u_{i}^{0}=\frac{1}{3}(u_{i}^{2}-6u_{i}^{1}+8U(x_{i}))\\ u_{0}^{n}=\frac{1}{3}(u_{2}^{n}-6u_{1}^{n})\\ u_{N_{x}+1}^{n}=\frac{1}{3}(u_{N_{x}-1}^{n}-6u_{N_{x}}^{n}) \end{array}$$

using second-order extrapolation. The loss function for the inverse problem consists of the residual and terms to impose the temperature measurements


(31) $$\begin{array}{rl} L(u,\theta ) & =\frac{1}{N_{x}N_{t}}\sum_{i=1}^{N_{x}}\sum_{n=1}^{N_{t}}(F_{i}^{n}(u,k_{\theta }){)}^{2}\\ & +\frac{w_{\mathrm{data}}^{2}}{N_{\mathrm{data}}}\sum_{i=1}^{N_{x}}\sum_{n=1}^{N_{t}}((u_{i}^{n}-{\tilde{U}}_{i}^{n})\chi_{i}^{n}{)}^{2}, \end{array}$$

where $w_{\mathrm{data}}=2$. We assume that the set of measurement points $\left({\tilde{x}}_{j},{\tilde{t}}_{j}\right)$ is a subset of the cell centers. If a cell (i,n) contains a measurement point *j*, then we set ${\tilde{U}}_{i}^{n}={\tilde{U}}_{j}$ and define a mask field $\chi_{i}^{n}=1$, otherwise $\chi_{i}^{n}=0$. For Newton’s method, the loss function additionally includes a damping term for the weights of the neural network


(32) $$L^{\text{Newton}}(u,\theta )=L(u,\theta )+\frac{w_{\theta }^{2}}{N_{\theta }}\sum_{i=1}^{N_{\theta }}(\theta_{i}-\theta_{i}^{*}{)}^{2},$$

where $w_{\theta }=1$, *θ* is a vector of all weights, and $\theta^{*}$ is a vector with the same weights but “frozen” so they are ignored in the linearization of the problem. This regularization makes the matrix in the linear system [13] nonsingular but does not affect the solution if the iteration converges.

**Fig. 4.**
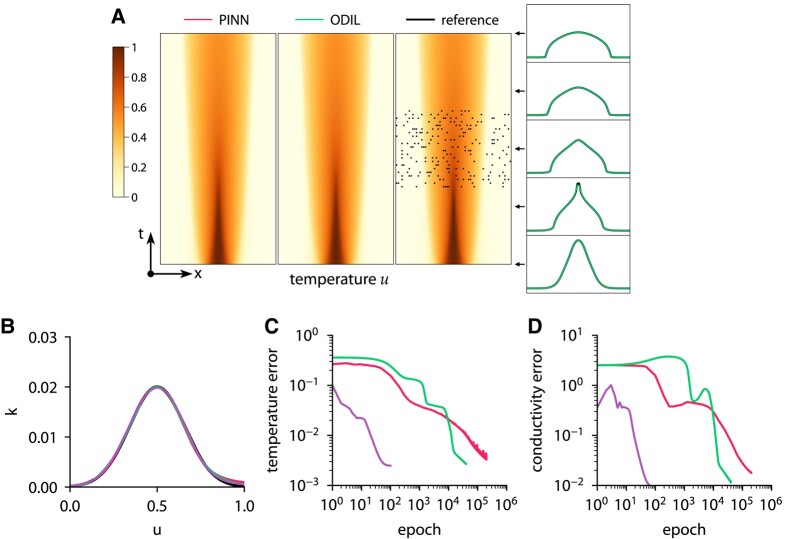
Inferring conductivity from temperature measurements using ODIL and PINN. Temperature field (A) and conductivity function (B) inferred by PINN with Adam 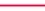, ODIL with Adam 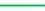, and ODIL with Newton 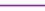 compared to reference 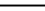. The reference solution is from forward problem solved using ODIL on a finer grid. The temperature measurements in 200 points (black dots) are taken from the reference solution. History of root-mean-square error in temperature (C) and conductivity (D).

**Fig. 5.**
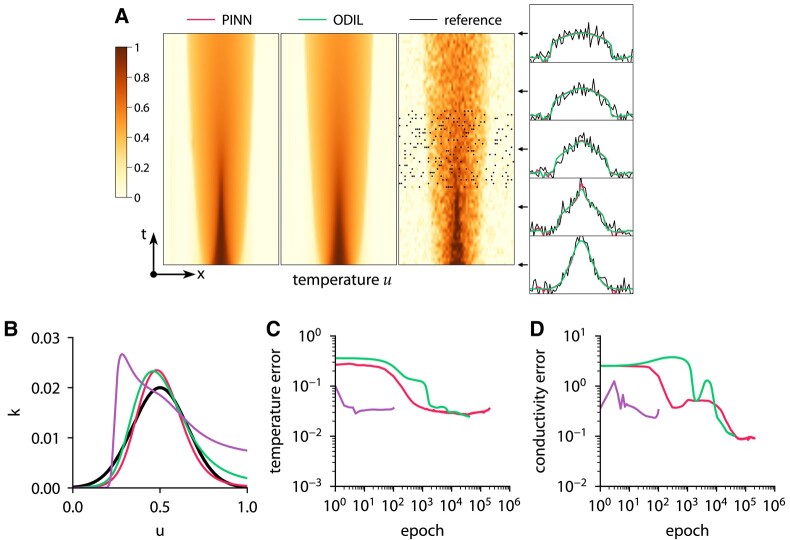
Inferring conductivity from noisy temperature measurements using ODIL and PINN. Temperature field (A) and conductivity function (B) inferred by PINN with Adam 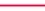, ODIL with Adam 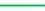, and ODIL with Newton 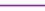 compared to reference 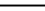. The reference solution is from forward problem solved using ODIL on a finer grid. The temperature measurements in 200 points (black dots) are taken from the reference solution perturbed by Gaussian noise with σ=0.1. History of root-mean-square error in temperature (C) and conductivity (D) relative to unperturbed reference solution.

To generate the temperature measurements and the reference solution, we specify the reference conductivity function as


(33) $$k(u)=0.02\;e^{-20(u-0.5{)}^{2}}$$

and solve the forward problem using ODIL on a grid of 256×256 cells. Then, we solve the inverse problem using PINN and ODIL and compare the results. To represent the unknown conductivity function k(u), we use a fully connected neural network of size 1×5×5×1, i.e. one input *u*, two hidden layers with five neurons in each layer and tanh activations, and its output *q* passed through $k=0.1/(1+e^{-q})$ to ensure that the conductivity is non-negative. To represent the temperature field u(x,t), we use a fully connected neural network of size 2×32×32×32×32×1 in PINN and a uniform grid of 64×64 cells in ODIL. The number of collocation points for PINN is 4,096 points inside the domain and 384 for the initial and boundary conditions. To solve the optimization problem with PINN and ODIL with use Adam with the learning rate set to 0.001, and also use Newton’s method with ODIL. Figure 4 shows the convergence history, the inferred temperature and conductivity. Both PINN and ODIL infer similar temperature fields and conductivity functions. The convergence history includes the root-mean-square error in the temperature field relative to the reference solution and the root-mean-square error in the conductivity function in the range u∈(0,1) with both quantities normalized by their maximum values in the reference solution. ODIL with Newton demonstrates the fastest convergence and takes about 25 iterations to achieve an error of 5% in the conductivity function. ODIL with Adam takes 13,000 iterations to achieve the same error. In contrast, PINN converges slower achieving the same error after 55,000 iterations. The execution time on one CPU core amounts to about 250 ms per iteration for PINN, 3.2 ms per iteration for ODIL with Adam, and 5,500 ms per iteration for ODIL with Newton. On a GPU, PINN takes 3.5 ms per iteration. Consistent with our previous observations, ODIL takes fewer iterations than PINN and each iteration is cheaper, which results in two to three orders of magnitude lower computational cost overall. Figure 5 shows the results if the temperature measurements are perturbed by Gaussian noise with standard deviation σ=0.1. The error is computed relative to the unperturbed reference solution. All methods achieve lower accuracy of recovering the conductivity function, ODIL with Newton is affected more by the added noise as the inferred conductivity has a larger error than that of ODIL and PINN optimized with Adam.

### Lid-driven cavity: forward problem

The lid-driven cavity problem is a standard test case (64) for numerical methods for the steady-state Navier–Stokes equations in two dimensions


(34) ux+vy=0,uux+vuy=−px+1Re(uxx+uyy),uvx+vvy=−py+1Re(vxx+vyy),

where u(x,y) and v(x,y) are the two velocity components and p(x,y) is the pressure. The problem is solved in a unit domain with no-slip boundary conditions. The upper boundary is moving to the right at a unit velocity while the other boundaries are stagnant. We solve this problem using ODIL and PINN. In ODIL, we use a finite volume discretization on a uniform Cartesian grid with the second-order upwind scheme for the momentum equations, Rhie-Chow interpolation (65) to prevent oscillations in the pressure field, and the deferred correction approach (66) that treats high-order discretization explicitly and low-order discretization implicitly to obtain an operator with a compact stencil and therefore reduce the size of the linear system for Newton’s method. In PINN, we use a fully connected network of size 2×64×64×64×64×64×3, with 400 collocation points on the boundaries, and inside the domain there are 10,000 points which are resampled every 100 epochs. The implementation of PINN is based on the DeepXDE framework (62). We refer to Figs. S6 and S7 for a hyperparameter study for PINN.

**Fig. 6.**
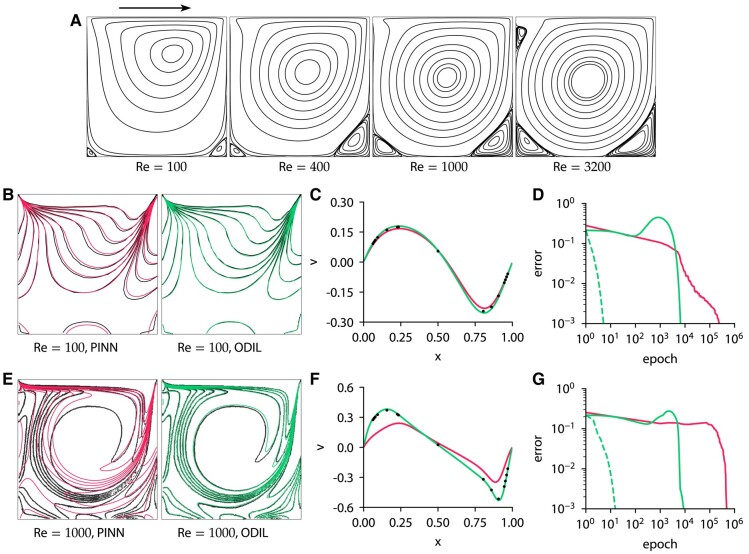
Lid-driven cavity problem solved using PINN and ODIL. A) Streamlines for Re=100,400,1000, and 3200 obtained using ODIL. The arrow indicates the direction of upper wall. B–D) Results for Re=100 obtained using PINN with L-BFGS-B 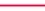 and ODIL with Newton 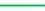. E–G) Same for Re=1000. B, E) Contours of vorticity compared to reference (64) 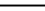. Reprinted with permission from Elsevier. C, F) Profiles of velocity *v* along the centerline y=0.5 compared to reference (64) 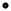. D, G) History of root-mean-square error in velocity *u* obtained using PINN with L-BFGS-B 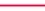, ODIL with Newton 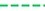, and ODIL with Adam 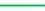. The error of PINN is relative to the solution after 500,000 epochs with L-BFGS-B. The error of ODIL is relative to the solution after 100 epochs with Newton.

Figure 6 shows the streamlines for values of Re between 100 and 3,200 obtained using ODIL with Newton on a 128×128 grid, as well as a convergence history of PINN with L-BFGS-B, ODIL with Adam, and ODIL with Newton. The learning rate of Adam is $10^{-3}$. The root-mean-square error in velocity *u* in ODIL is computed relative to the solution after 100 epochs with Newton. The error of PINN is measured relative to the solution after 500,000 epochs with L-BFGS-B. To get an idea about the convergence speed of each method, we measure how many epochs it takes to reach an error of 1%. At Re=100, ODIL with Adam takes 5,500 epochs and PINN takes 18,000 epochs. At Re=1000, ODIL with Adam takes 6,050 epochs and PINN formally takes 420,000 epochs, but that indicates that the PINN optimization has not converged after 500,000 epochs. In contrast, ODIL with Newton takes three iterations at Re=100 and eight iterations at Re=1000, showing that Newton’s method converges much faster, but at the cost of more expensive iterations. ODIL with Newton takes 2,200 ms per epoch, ODIL with Adam takes 8 ms on one CPU core, and PINN takes 30 ms on a GPU Nvidia A100. The results shown in Fig. 6 as contour plots of vorticity and profiles of vertical velocity agree well with reference numerical data (64) for both methods at Re=100, but PINN at Re=1000 fails to reproduce the reference data.

### Lid-driven cavity: flow reconstruction

**Fig. 7.**
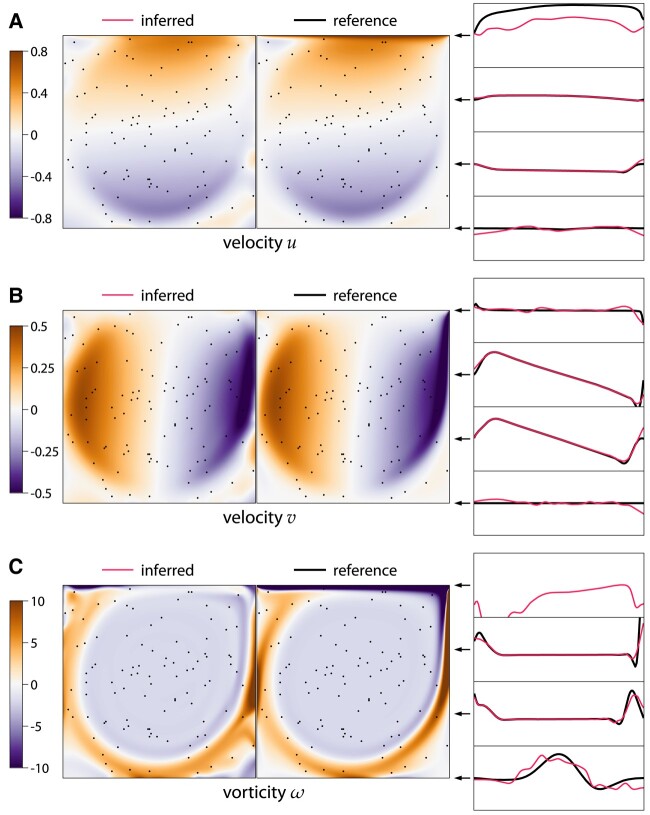
Lid-driven cavity flow at Re=3200 reconstructed using ODIL from velocity measurements in 100 points (black dots) without boundary conditions for velocity. Inferred and reference velocity *u* (A), velocity *v* (B), and vorticity *ω* (C).

Here, we solve the same equations [34] as in the forward problem, but we impose the known velocity values $u(x_{j})={\tilde{u}}_{j}$ in a finite set of measurement points $x_{i}$ for $j=1,\ldots ,N_{\mathrm{data}}$ with $N_{\mathrm{data}}=100$. However, we remove the no-slip boundary conditions and replace them with linear extrapolation, turning this problem into a flow reconstruction problem. The loss function is the same as in the forward problem, but the known velocity values are imposed through reparameterization of the velocity field, which takes the known value in cells containing a measurement point, and an unknown value otherwise. The problem is solved on a grid of 128×128 cells and the reference velocity field is obtained from the forward problem. Figure 7 shows the results of reconstruction from 100 points.

### Measure of flow complexity

Based on the flow reconstruction from velocity measurements using ODIL in the previous case, we introduce a measure of flow complexity. Consider a velocity field $u_{\mathrm{ref}}$ that satisfies the Navier–Stokes equations. First, we define the minimal reconstruction error given *K* points


(35) $$E_{\min}(K)=\min_{|X|=K}\|u_{\mathrm{ref}}-u(X)\|,$$

where u(X) is the velocity field reconstructed from points *X* and the minimum is taken over all sets of *K* points. Then, we define a measure of flow complexity for a given accuracy ε>0 as the minimal number of points required to achieve that accuracy


(36) $$K_{\min}(\varepsilon )=\{K\;|\;E_{\min}(K)<\varepsilon \}.$$

**Fig. 8.**
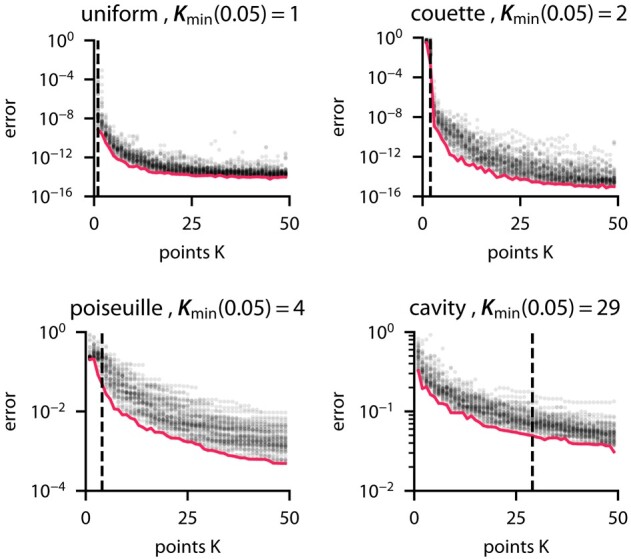
Fluid flow reconstruction error depending on the number of measurement points for various flows: uniform, Couette flow, Poiseuille flow, and lid-driven cavity. The dots show samples of random sets of *K* points and the minimum over them is an estimate of the minimal reconstruction error $E_{\min}(K)$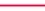.

To illustrate this complexity measure, we consider four types of flow: uniform velocity, Couette flow (linear profile), Poiseuille flow (parabolic profile), and the flow in a lid-driven cavity. The reference velocity fields in the first three cases are chosen once at an arbitrary orientation and such that the maximum velocity magnitude is unity. As in the previous case, we do not impose any boundary conditions for the velocity, replacing them with linear extrapolation. However, to generate the samples faster, we use a smaller N×N grid with N=64 and optimize using Newton’s method. Newton’s method with ODIL requires adding a regularization term, for which we choose a discretization of


(37) $$k_{\mathrm{reg}}^{2}(\|u_{xx}{\|}^{2}+\|u_{yy}{\|}^{2}+\|v_{xx}{\|}^{2}+\|v_{yy}{\|}^{2})$$

with $k_{\mathrm{reg}}=10^{-3}$. Figure 8 shows the values of E(K) and $K_{\min}(0.05)$. The error is the mean absolute difference between the inferred and reference solutions summed over both velocity components. As expected, the uniform flow takes 1 point to reconstruct while adding higher order terms to the velocity increases the number of points. Finally, 29 points are required to reach an error of 0.05 for the lid-driven cavity flow.

### Inferring body shape from velocity in two dimensions

Here, we consider a 2D inverse problem of inferring the shape of a body from measurements of the flow velocity around the body. The model consists of the steady-state Navier–Stokes equations with penalization terms to impose the no-slip conditions on the body (67)


(38) ux+vy=0,(1−χ)(uux+vuy+px−DRe(uxx+uyy))+λχu=0,(1−χ)(uvx+vvy+py−DRe(vxx+vyy))+λχv=0,

where *λ* is a penalization parameter and *D* is a characteristic length of the body. The shape of the body is described by the body fraction χ(x,y) which takes values χ=1 inside the body and χ=0 outside. The problem is solved in the domain [0,2]×[0,1] with the inlet conditions u=1 and v=0 at x=0, outlet at x=2, and free-slip walls at y=0 and y=1. The discretization of the Navier–Stokes equations follows the one used for the lid-driven cavity problem. The forward problem is to find the velocities *u* and *v* and pressure *p* given equations [38] with boundary conditions and a prescribed body fraction *χ*. The inverse problem is to find the velocities *u* and *v*, pressure *p*, and body fraction *χ* given equations [38] with boundary conditions and values of *u* and *v* in a set of measurement points.

To solve the inverse problem using ODIL, we formulate it as minimization of the loss function in terms of the unknown fields: velocities *u* and *v*, pressure *p*, and a level-set function φ. The problem is solved on a 2N×N grid with N=64. The known velocity values are imposed through reparameterization of the velocity field, which takes the known value in cells containing a measurement point, and an unknown value otherwise. The loss function consists of the residuals of the governing equations [38] and a term with the eikonal equation for the level-set function


(39) $$k_{\phi }^{2}\||\nabla \varphi {|}^{2}-1{\|}_{2}^{2}$$

discretized using an upwind scheme (68). The parameter decays as $k_{\phi }=\text{max}(1,10\times (0.5{)}^{s/1,000})$ with epoch *s*, and the penalization parameter is set to λ=10. The level-set function determines the body fraction as


(40) $$\chi =\text{max}\left(0,\min\left(1,\frac{1}{2}+\frac{\phi }{4\Delta x}\right)\right).$$

**Fig. 9.**
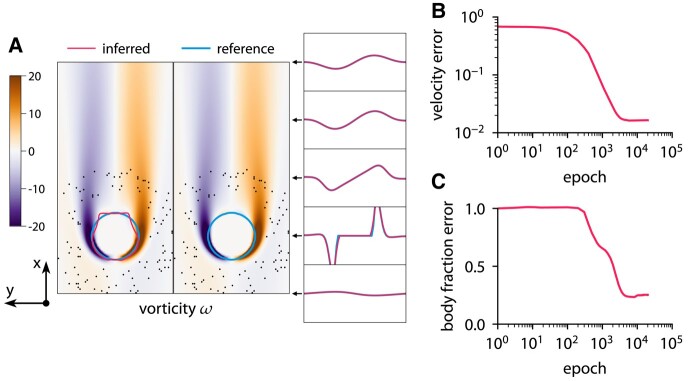
Inferring body shape from velocity measurements for a flow past a circle at Re=60 using ODIL. The reference solution is from the forward problem solved using ODIL. A) Inferred 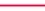 and reference 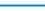 vorticity *ω* overlapped by contours of body fraction χ=0.5. The velocity measurements are imposed in 100 points (black dots) chosen at a distance from the body. B) History of root-mean-square in velocity *u*. C) History of root-mean-square in body fraction *χ* normalized by the mean of reference. See Fig. S13 for visualizations of velocity *u*, velocity *v*, body fraction *χ*, and level-set function *ϕ*.

**Fig. 10.**
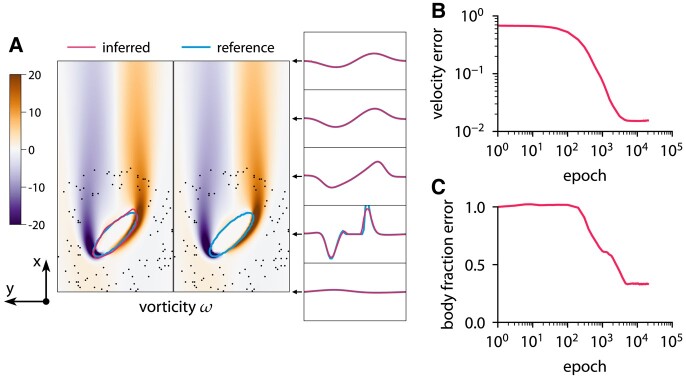
Inferring body shape from velocity measurements for a flow past an ellipse at Re=60 using ODIL. The reference solution is from the forward problem solved using ODIL. A) Inferred 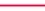 and reference 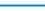 vorticity *ω* overlapped by contours of body fraction χ=0.5. The velocity measurements are imposed in 100 points (black dots) chosen at a distance from the body. B) History of root-mean-square in velocity *u*. C) History of root-mean-square in body fraction *χ* normalized by the mean of reference. See Fig. S14 for visualizations of velocity *u*, velocity *v*, body fraction *χ*, and level-set function *ϕ*.

**Fig. 11.**
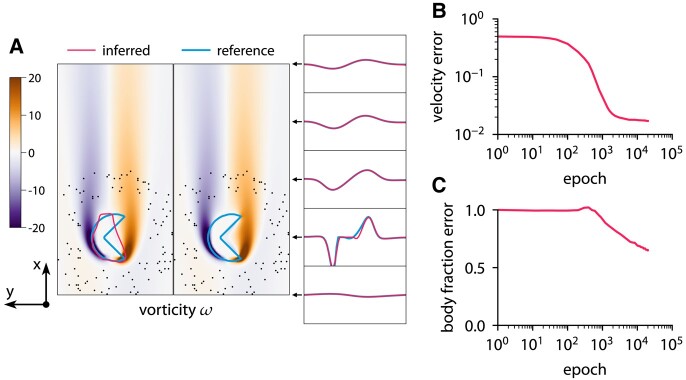
Inferring body shape from velocity measurements for a flow past a nonconvex body at Re=60 using ODIL. The reference solution is from the forward problem solved using ODIL. A) Inferred 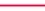 and reference 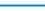 vorticity *ω* overlapped by contours of body fraction χ=0.5. The velocity measurements are imposed in 100 points (black dots) chosen at a distance from the body. B) History of root-mean-square in velocity *u*. C) History of root-mean-square in body fraction *χ* normalized by the mean of reference. See Fig. S15 for visualizations of velocity *u*, velocity *v*, body fraction *χ*, and level-set function *ϕ*.

This approach with the level-set function ensures that the interface is diffused over a constant number of cells. The reference data are obtained from the forward problem. The chosen grid is sufficiently fine to resolve the flow based on the grid refinement study in Fig. S11. Figures 9–11 show the results of inference for three different bodies: a circle, an ellipse, and a nonconvex body. The characteristic length of the body is D=0.4, and the Reynolds number is Re=60. To solve the optimization problem, we use Adam with a learning rate of 0.001. The initial guess is u=1, v=0, p=0, and the level-set function


(41) $$\phi (x,y)=0.02-\sqrt{(x-0.5{)}^{2}+(y-0.5{)}^{2}}$$

describing a small circle. The optimization takes 20,000 iterations and completes in about 3 min on one CPU core. ODIL recovers the reference shape from velocity measurements in 100 points if the shape is convex. However, we observe that the method does not infer nonconvex shapes and instead finds a convex shape that matches the velocity measurements.

### Inferring body shape from velocity in three dimensions

Here, we consider a 3D inverse problem of inferring the shape of a body from measurements of the flow velocity around the body. The model consists of the steady-state Navier–Stokes equations with penalization terms to impose the no-slip conditions on the body (67)


(42) ∇⋅u=0,(1−χ)((u⋅∇)u+∇p−DRe∇2u)+λχu=0,

where *λ* is a penalization parameter and *D* is a characteristic length of the body. The shape of the body is described by the body fraction χ(x) which takes values χ=1 inside the body and χ=0 outside. The problem is solved in the domain [0,2]×[0,1]×[0,1] with the inlet condition u=(1,0,0) at x=0, outlet condition p=0 at x=2, and free-slip walls on the other boundaries. The discretization of the Navier–Stokes equations follows the one used for the lid-driven cavity problem. The forward problem is to find the velocity u and pressure *p* that satisfy Eqs. (42) given a prescribed body fraction *χ*. The inverse problem is to find the velocity u, pressure *p*, and body fraction *χ* that satisfy Eqs. (42) such that the velocity field takes known values $u(x_{i})=u_{i}$ in a finite set of *N* measurement points $x_{i}$ for i=1,…,N.

To solve the inverse problem using ODIL, we formulate it as minimization of the loss function in terms of the unknown fields: velocity u, pressure *p*, and body fraction $\hat{\chi }$. Here, $\hat{\chi }$ is a transformed body fraction defined as $\chi =1/(1+e^{-(\hat{\chi }+5)})$, so that during the optimization the body fraction *χ* only takes values between 0 and 1. The loss function is a sum of the residuals of equations (42) and terms to impose the reference data. We do not include any regularization terms. The penalization parameter is set to λ=1. The problem is solved on a 129×65×65 grid, and the reference data are obtained from the forward problem. The characteristic length of the body is D=0.4 and the Reynolds number is Re=60. To solve the optimization problem, we use L-BFGS (69) implemented in TensorFlow Probability (70). The initial guess is u=(1,0,0) for the velocity, p=0 for the pressure, and $\hat{\chi }=0$ for the transformed body fraction. According to the above transformation, the corresponding initial guess for the body fraction is $\chi =1/(1+e^{5})$. We terminate the algorithm after 10,000 epochs for the forward problem and 20,000 epochs for the inverse problem.

**Fig. 12.**
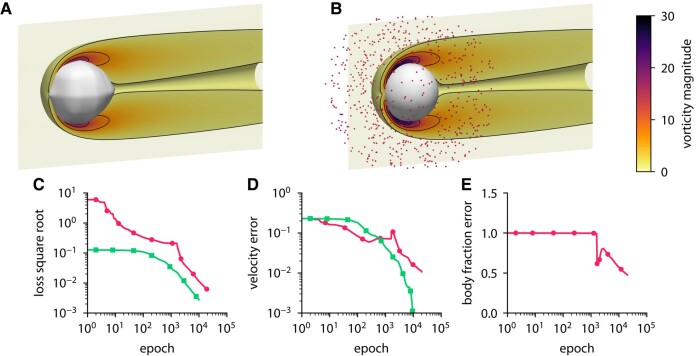
Inferring body shape from velocity measurements for a flow past a sphere using ODIL. A, B) Inferred (A) and reference (B) body shape and contours of vorticity magnitude. The velocity measurements are imposed in 684 points (red dots) chosen at a distance from the body. C–E) History of the square root of the loss (C), root-mean-square error in *x*-velocity (D), and body fraction *χ* normalized by the mean of reference (E) for the inverse problem 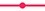 and forward problem 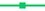. The error is relative to the solution of the forward problem after 10,000 epochs.

**Fig. 13.**
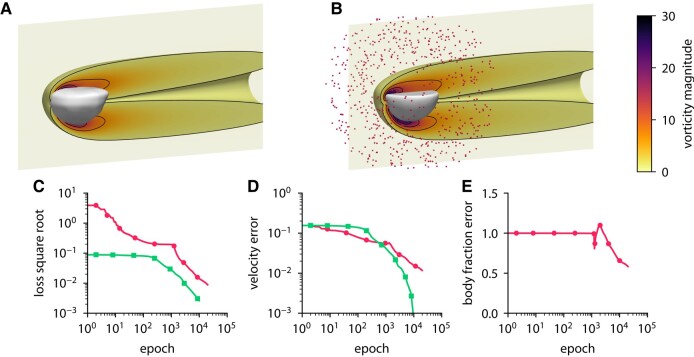
Inferring body shape from velocity measurements for a flow past a hemisphere using ODIL. A, B) Inferred (A) and reference (B) body shape and contours of vorticity magnitude. The velocity measurements are imposed in 684 points (red dots) chosen at a distance from the body. C–E) History of the square root of the loss (C), root-mean-square error in *x*-velocity (D), and body fraction *χ* normalized by the mean of reference (E) for the inverse problem 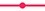 and forward problem 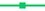. The error is relative to the solution of the forward problem after 10,000 epochs.

Figures 12 and 13 show the results of the inference from 684 measurement points for two different bodies: a sphere and a hemisphere. The convergence history includes the velocity error and the body fraction error which are defined relative to the solution of the forward problem. In both cases, ODIL recovers a body shape that qualitatively agrees with the reference, although the relative error in the body fraction field amounts to 50%, so the inferred body volume is larger. On a GPU Nvidia A100, the forward problem with a sphere takes 53 min in total and 320ms per epoch, while the inverse problem takes 122 min in total and 366ms per epoch. We note that solving the same inverse problem on a finer grid of 257×129×129 cells takes 132 min in total and 400ms per epoch. Therefore, an eightfold increase in the number of grid cells will lead to a minor additional cost in the execution time of 8% since the GPU operates more efficiently with larger arrays.

## Conclusion

We introduce the ODIL framework for solving inverse problems for PDEs by casting their discretization as an optimization problem and applying optimization techniques that are widely available in machine-learning software. The concept of casting the PDE as is closely related to the neural network formulations proposed by (15–17) and recently revived as PINNs. However, the fact that we use the discrete approximation of the equations allows for ODIL to be orders of magnitude more efficient in terms of computational cost and accuracy compared to the PINN for which complex flow problems “remain elusive” (71).

We remark that the error of PINN typically scales as a square root of the number of training points regardless of the space dimensionality (25), which can be beneficial for high-dimensional problems (72, 73). However, a recent attempt (36) to apply PINN for simulating an open quantum system has proven unsuccessful, outperformed by the Q-Flow method based on normalizing flows proposed therein. In ODIL, the grid-based discretization may suffer from the curse of dimensionality as the error of a *p*-order accurate method with *N* grid points scales as $O(N^{\;p/d})$ in a *d*-dimensional space. To solve high-dimensional problems with ODIL, we suggest the use of use of sparse grids (74–76) that introduce a hierarchical basis so that the error scales as $O(N^{p}(\log\;N{)}^{d})$ with the number of space dimensions. Another approach is to use a grid in some of the dimensions (e.g. spatial dimensions) and discretize the remaining dimensions with neural networks or basis functions such as spherical harmonics. The latter approach has outperformed fully connected networks by two orders of magnitude on problems of view synthesis in computer graphics (77). The treatment of complex geometries with PINNs is equivalent to treatments deployed in particle methods, using globally interpolating kernels (1). as the collocation points can be positioned arbitrarily. For ODIL, we have demonstrated here the use of the penalization method for the flow past a body. Another possibility is to use an unstructured grid fitted to the complex boundaries or even ideas from particle methods.

We have presented the applications of ODIL to several forward and inverse problems involving PDEs, including the Poisson equation, the heat equation, the advection equation, and the Navier–Stokes equations. In most cases, the discrete loss has been formulated using second-order accurate finite volume discretizations and incorporate exactly the boundary and initial conditions through extrapolation. We emphasize that the primary scope of ODIL is in solving ill-posed and inverse problems. For forward, and in particular time-dependent problems, a multitude of classical solvers based on marching in time are far more efficient than PINNs and ODIL since they do not require storing all the time steps as unknowns.

The ideas and results presented in this article suggest that ODIL, and even more the ideas it entails, can serve as inspiration for advancing the solution of inverse problems. We remark that while ODIL is shown to vastly outperform PINNs, its development was motivated by the widespread use of PINNs and their treatment of equations and data. We believe that there are many promising venues at such interfaces (and competitions) of machine learning and scientific computing for solving inverse problems using physics and data informed methodologies. Ongoing works using ODIL include tracking the evolution of brain tumors from medical images (78), the derivation of material properties for specific heat conduction and the identification of submerged obstacles from surface information.

## Supplementary Material

pgae005_Supplementary_Data

## Data Availability

The source code of the implementation of the method is available at https://github.com/cselab/odil along with examples and instructions to reproduce the results.

## Preprints

A preprint of this article is published at arXiv:2205.04611.

## References

[1] Koumoutsakos P . 2005. Multiscale flow simulations using particles. Annu Rev Fluid Mech. 37(1):457–487.

[2] LeVeque RJ . 2007. Finite difference methods for ordinary and partial differential equations: steady-state and time-dependent problems. Philadelphia (PA): SIAM.

[3] Zienkiewicz OC , Taylor RL, Zhu JZ. 2005. The finite element method: its basis and fundamentals. Oxford: Elsevier.

[4] Brunton SL , Kutz JN. 2019. Data-driven science and engineering: machine learning, dynamical systems, and control. Cambridge: Cambridge University Press.

[5] Cui T , Marzouk YM, Willcox KE. 2015. Data-driven model reduction for the Bayesian solution of inverse problems. Int J Numer Methods Eng. 102(5):966–990.

[6] Ghattas O , Willcox K. 2021. Learning physics-based models from data: perspectives from inverse problems and model reduction. Acta Numer. 30:445–554.

[7] Karniadakis GE , Kevrekidis IG, Lu L, Perdikaris P, Wang S, Yang L. 2021. Physics-informed machine learning. Nat Rev Phys. 3(6):422–440.

[8] Boche H , Fono A, Kutyniok G. 2023. Limitations of deep learning for inverse problems on digital hardware. IEEE Trans Inf Theory. 69(12):7887.

[9] Gunzburger MD . 2002. Perspectives in flow control and optimization. Philadelphia (PA): SIAM.

[10] Lewis JM , Lakshmivarahan S, Dhall S. 2006. Dynamic data assimilation: a least squares approach. Vol. 13. Cambridge: Cambridge University Press.

[11] Fleet D , Weiss Y. 2006. Optical flow estimation. In: Paragios N, Chen Y, Faugeras O, editors. Handbook of mathematical models in computer vision. Boston (MA): Springer. p. 237–257.

[12] Ljung L . 1999. System identification: theory for the user. 2nd ed. USA: Prentice Hall PTR.

[13] Bar-Sinai Y , Hoyer S, Hickey J, Brenner MP. 2019. Learning data-driven discretizations for partial differential equations. Proc Natl Acad Sci U S A. 116(31):15344–15349. 31311866 10.1073/pnas.1814058116 PMC6681734

[14] Vlachas PR , *et al*. 2020. Backpropagation algorithms and reservoir computing in recurrent neural networks for the forecasting of complex spatiotemporal dynamics. Neural Netw. 126:191–217. 32248008 10.1016/j.neunet.2020.02.016

[15] Dissanayake M , Phan-Thien N. 1994. Neural-network-based approximations for solving partial differential equations. Commun Numer Methods Eng. 10(3):195–201.

[16] van Milligen BP , Tribaldos V, Jiménez J. 1995. Neural network differential equation and plasma equilibrium solver. Phys Rev Lett. 75(20):3594–3597. 10059679 10.1103/PhysRevLett.75.3594

[17] Lagaris IE , Likas A, Fotiadis DI. 1998. Artificial neural networks for solving ordinary and partial differential equations. IEEE Trans Neural Netw. 9(5):987–1000. 18255782 10.1109/72.712178

[18] Gicquel N , Anderson J, Kevrekidis I. 1998. Noninvertibility and resonance in discrete-time neural networks for time-series processing. Phys Lett A. 238(1):8–18.

[19] Rico-Martinez R , Kevrekidis IG. 1993. Continuous time modeling of nonlinear systems: a neural network-based approach. In: IEEE International Conference on Neural Networks. IEEE. p. 1522–1525.

[20] Quito Jr M , Monterola C, Saloma C. 2001. Solving *N*-body problems with neural networks. Phys Rev Lett. 86(21):4741–4744. 11384337 10.1103/PhysRevLett.86.4741

[21] Milano M , Koumoutsakos P. 2002. Neural network modeling for near wall turbulent flow. J Comput Phys. 182(1):1–26.

[22] Raissi M , Perdikaris P, Karniadakis GE. 2019. Physics-informed neural networks: a deep learning framework for solving forward and inverse problems involving nonlinear partial differential equations. J Comput Phys. 378:686–707.

[23] Basir S , Senocak I. 2022. Critical investigation of failure modes in physics-informed neural networks. In: AIAA SCITECH 2022 Forum, San Diego (CA), USA. p. 2353.

[24] Krishnapriyan A , Gholami A, Zhe S, Kirby R, Mahoney MW. 2021. Characterizing possible failure modes in physics-informed neural networks. Adv Neural Inf Process Syst. 34:26548–26560.

[25] Mishra S , Molinaro R. 2022. Estimates on the generalization error of physics-informed neural networks for approximating a class of inverse problems for PDES. IMA J Numer Anal. 42(2):981–1022.

[26] Mattey R , Ghosh S. 2022. A novel sequential method to train physics informed neural networks for Allen Cahn and Cahn Hilliard equations. Comput Methods Appl Mech Eng. 390:114474.

[27] Mistani PA , Pakravan S, Ilango R, Gibou F. 2023. JAX-DIPS: neural bootstrapping of finite discretization methods and application to elliptic problems with discontinuities. J Comput Phys. 493:112480.

[28] Baydin AG , Pearlmutter BA, Radul AA, Siskind JM. 2018. Automatic differentiation in machine learning: a survey. J March Learn Res. 18:1–43.

[29] Bettencourt J , Johnson MJ, Duvenaud D. 2019. Taylor-mode automatic differentiation for higher-order derivatives in JAX. In: Program Transformations for ML Workshop at NeurIPS 2019.

[30] Basir S , Senocak I. 2022. Physics and equality constrained artificial neural networks: application to forward and inverse problems with multi-fidelity data fusion. J Comput Phys. 463:111301.

[31] Dong S , Ni N. 2021. A method for representing periodic functions and enforcing exactly periodic boundary conditions with deep neural networks. J Comput Phys. 435:110242.

[32] Jagtap AD , Kawaguchi K, Karniadakis GE. 2020. Adaptive activation functions accelerate convergence in deep and physics-informed neural networks. J Comput Phys. 404:109136. 10.1098/rspa.2020.0334 PMC7426042 32831616

[33] McGreivy N , Hakim A. 2023. Invariant preservation in machine learned PDE solvers via error correction, arXiv, arXiv:2303.16110, preprint: not peer reviewed.

[34] Sukumar N , Srivastava A. 2022. Exact imposition of boundary conditions with distance functions in physics-informed deep neural networks. Comput Methods Appl Mech Eng. 389:114333.

[35] Chuang P-Y , Barba LA. 2022. Experience report of physics-informed neural networks in fluid simulations: pitfalls and frustration, arXiv, arXiv:2205.14249, preprint: not peer reviewed.

[36] Dugan OM , Lu PY, Dangovski R, Luo D, Soljacic M. 2023. Q-flow: generative modeling for differential equations of open quantum dynamics with normalizing flows. In: International Conference on Machine Learning, Honolulu (HI), USA. PMLR. p. 8879–8901.

[37] Grossmann TG , Komorowska UJ, Latz J, Schönlieb C-B. 2023. Can physics-informed neural networks beat the finite element method?, arXiv, arXiv:2302.04107, preprint: not peer reviewed. 10.1093/imamat/hxae011 PMC11197852 38933736

[38] Wang Z , Meng X, Jiang X, Xiang H, Karniadakis GE. 2023. Solution multiplicity and effects of data and eddy viscosity on Navier–Stokes solutions inferred by physics-informed neural networks, arXiv, arXiv:2309.06010, preprint: not peer reviewed.

[39] van Leeuwen T , Herrmann FJ. 2015. A penalty method for PDE-constrained optimization in inverse problems. Inverse Probl. 32(1):015007.

[40] Schlottbom M , Pietschmann J-F. 2022. Data-driven gradient flows. Electron Trans Numer Anal. 57:193–215.

[41] Kaltenbacher B . 2016. Regularization based on all-at-once formulations for inverse problems. SIAM J Numer Anal. 54(4):2594–2618.

[42] Amos B , Kolter JZ. 2017. Optnet: differentiable optimization as a layer in neural networks. In: International Conference on Machine Learning, Sydney, Australia. PMLR. p. 136–145.

[43] Liang J , Lin M, Koltun V. 2019. Differentiable cloth simulation for inverse problems. Adv Neural Inf Process Syst. 32:772–781.

[44] List B , Chen L-W, Thuerey N. 2022. Learned turbulence modelling with differentiable fluid solvers: physics-based loss functions and optimisation horizons. J Fluid Mech. 949:A25.

[45] Wandel N , Weinmann M, Klein R. 2021. Learning incompressible fluid dynamics from scratch - towards fast, differentiable fluid models that generalize. In: International Conference on Learning Representations.

[46] Betts JT . 2010. Practical methods for optimal control and estimation using nonlinear programming. Philadelphia (PA): SIAM.

[47] Bock HG . 1983. Recent advances in parameteridentification techniques for ode. In: Numerical Treatment of Inverse Problems in Differential and Integral Equations: Proceedings of an International Workshop, Heidelberg, Fed. Rep. of Germany, August 30-September 3, 1982. Springer. p. 95–121.

[48] Mistani P , Pakravan S, Ilango R, Choudhry S, Gibou F. 2022. Neuro-symbolic partial differential equation solver. In: Neural Information Processing Systems, Machine Learning and the Physical Sciences Workshop, New Orleans, LA.

[49] Rahaman N , *et al*. 2019. On the spectral bias of neural networks. In: International Conference on Machine Learning, Long Beach, CA. PMLR. p. 5301–5310.

[50] Pakravan S , Mistani PA, Aragon-Calvo MA, Gibou F. 2021. Solving inverse-PDE problems with physics-aware neural networks. J Comput Phys. 440:110414.

[51] Kingma DP , Ba J. 2014. Adam: a method for stochastic optimization, arXiv, arXiv:1412.6980, preprint: not peer reviewed.

[52] Zhu C , Byrd RH, Lu P, Nocedal J. 1997. Algorithm 778: L-BFGS-B: Fortran subroutines for large-scale bound-constrained optimization. ACM Trans Math Softw (TOMS). 23(4):550–560.

[53] Abadi M , *et al*. TensorFlow: large-scale machine learning on heterogeneous systems, 2015. Software available from tensorflow.org.

[54] Demmel JW , Eisenstat SC, Gilbert JR, Li XS, Liu JWH. 1999. A supernodal approach to sparse partial pivoting. SIAM J Matrix Anal Appl. 20(3):720–755.

[55] Virtanen P , *et al*. 2020. SciPy 1.0: fundamental algorithms for scientific computing in Python. Nat Methods. 17(3):261–272. 32015543 10.1038/s41592-019-0686-2 PMC7056644

[56] Bell N , Olson LN, Schroder J. 2022. PyAMG: algebraic multigrid solvers in python. J Open Source Softw. 7(72):4142.

[57] Coleman TF , Moré JJ. 1983. Estimation of sparse Jacobian matrices and graph coloring problems. SIAM J Numer Anal. 20(1):187–209.

[58] Nocedal J , Wright SJ. 1999. Numerical optimization. New York (NY): Springer.

[59] Trottenberg U , Oosterlee CW, Schuller A. 2000. Multigrid. London: Academic Press.

[60] Naumov M , *et al*. 2015. AmgX: a library for GPU accelerated algebraic multigrid and preconditioned iterative methods. SIAM J Sci Comput. 37(5):S602–S626.

[61] Karnakov P , Litvinov S, Koumoutsakos P. 2023. Flow reconstruction by multiresolution optimization of a discrete loss with automatic differentiation. Eur Phys J E. 46(7):59. 37486579 10.1140/epje/s10189-023-00313-7

[62] Lu L , Meng X, Mao Z, Karniadakis GE. 2021. DeepXDE: a deep learning library for solving differential equations. SIAM Rev. 63(1):208–228.

[63] Mang A , Gholami A, Davatzikos C, Biros G. 2018. PDE-constrained optimization in medical image analysis. Optim Eng. 19(3):765–812.

[64] Ghia U , Ghia KN, Shin C. 1982. High-Re solutions for incompressible flow using the Navier–Stokes equations and a multigrid method. J Comput Phys. 48(3):387–411.

[65] Rhie CM , Chow W-L. 1983. Numerical study of the turbulent flow past an airfoil with trailing edge separation. AIAA J. 21(11):1525–1532.

[66] Ferziger JH , Peric M. 2012. Computational methods for fluid dynamics. Cham: Springer Science & Business Media.

[67] Angot P , Bruneau C-H, Fabrie P. 1999. A penalization method to take into account obstacles in incompressible viscous flows. Numer Math. 81(4):497–520.

[68] Osher S , Sethian JA. 1988. Fronts propagating with curvature-dependent speed: algorithms based on Hamilton–Jacobi formulations. J Comput Phys. 79(1):12–49.

[69] Liu DC , Nocedal J. 1989. On the limited memory BFGS method for large scale optimization. Math Program. 45(1-3):503–528.

[70] Dillon JV , *et al*. 2017. Tensorflow distributions, arXiv, arXiv:1711.10604, preprint: not peer reviewed.

[71] Wang S , Sankaran S, Perdikaris P. 2022. Respecting causality is all you need for training physics-informed neural networks, arXiv, arXiv:2203.07404, preprint: not peer reviewed.

[72] Kawaguchi S , Murakami T. 2022. Physics-informed neural networks for solving the Boltzmann equation of the electron velocity distribution function in weakly ionized plasmas. Jpn J Appl Phys. 61(8):086002.

[73] Mishra S , Molinaro R. 2021. Physics informed neural networks for simulating radiative transfer. J Quant Spectrosc Radiat Transf. 270:107705.

[74] Garcke J . 2012. Sparse grids in a nutshell. In: Garcke J, Griebel M, editors. Sparse grids and applications. Vol. 88. Berlin: Springer. p. 57–80.

[75] Nobile F , Tempone R, Webster CG. 2008. A sparse grid stochastic collocation method for partial differential equations with random input data. SIAM J Numer Anal. 46(5):2309–2345.

[76] Smolyak SA . 1963. Quadrature and interpolation formulas for tensor products of certain classes of functions. Dokl Akad Nauk SSSR. 148(5):1042–1045.

[77] Fridovich-Keil S , et al 2022. Plenoxels: radiance fields without neural networks. In: Proceedings of the IEEE/CVF Conference on Computer Vision and Pattern Recognition, New Orleans, LA. p. 5501–5510.

[78] Balcerak M , *et al*. 2023. Individualizing glioma radiotherapy planning by optimization of a data and physics informed discrete loss, arXiv, arXiv:2312.05063, preprint: not peer reviewed.

