# Supplementary material for: Solving inverse problems in physics by optimizing a discrete loss: Fast and accurate learning without neural networks
Source: PNAS Nexus. 2024 Jan 11;3(1):pgae005. doi: 10.1093/pnasnexus/pgae005 (PMC10799659; doi:10.1093/pnasnexus/pgae005)
Supplement: pgae005_Supplementary_Data [file pgae005_supplementary_data.pdf]

Supplementary Material for

# Solving inverse problems in physics by optimizing a discrete loss: fast and accurate learning without neural networks

Petr Karnakov, Sergey Litvinov, Petros Koumoutsakos*

*Corresponding author. Email: petros@seas.harvard.edu

**This PDF file includes:**

Supplementary Text
Figs. S1 to S15

**Supplementary Text**

Algebraic equation

We consider an algebraic equation $\mathbf{F}(\mathbf{x}) = \mathbf{0}$ with two unknowns:

$$\mathbf{F}(\mathbf{x}) = \begin{pmatrix} x - 2 \\ x - y^2 + xy \end{pmatrix}, \quad \mathbf{x} = \begin{pmatrix} x \\ y \end{pmatrix}. \tag{1}$$

We solve it in the context of ODIL using as optimization algorithms, the gradient descent and Newton's method. ODIL reformulates this problem as minimization of a loss function

$$\mathcal{L}(\mathbf{x}) = \tfrac{1}{2}\mathbf{F}(\mathbf{x}) \cdot \mathbf{F}(\mathbf{x}). \tag{2}$$

One iteration of the gradient descent method is

$$\mathbf{x}^{s+1} = \mathbf{x}^s - \eta(\nabla \mathbf{F}^s)^T \mathbf{F}^s, \tag{3}$$

and one iteration of Newton's method is

$$\nabla \mathbf{F}^s(\mathbf{x}^{s+1} - \mathbf{x}^s) = \mathbf{F}^s, \tag{4}$$

where $\mathbf{F}^s = \mathbf{F}(\mathbf{x}^s)$, $\nabla \mathbf{F}^s$ is the Jacobian of $\mathbf{F}$ at point $\mathbf{x}^s$, and $\eta = 0.2$ is a constant parameter.

Fig. S1 shows the trajectory and convergence history of both algorithms started from an initial guess $\mathbf{x}^0 = (1, 1)^T$. Newton's method converges faster and maintains the exact solution $x = 2$ of the first equation throughout the optimization.

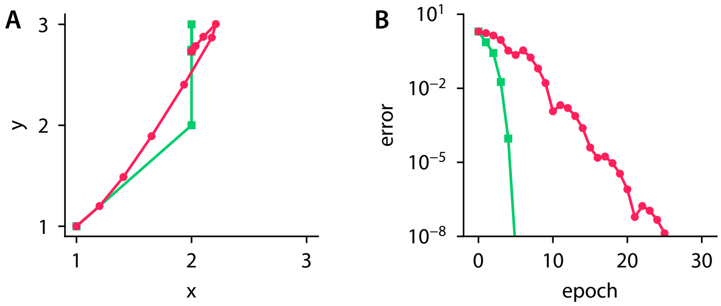


**Fig. S1.** Algebraic equation with two unknowns solved using gradient descent —•— and Newton —▪—. A) Trajectory of each method starting from the initial guess $(x, y) = (1, 1)$. B) History of the $L_2$ error.

One-dimensional finite difference equation

Here we examine the performance of two optimization algorithms, namely the gradient descent and Newton's method, on a boundary value problem for a one-dimensional finite difference equation. The unknown field is $u_i$ for $i = 1, \dots, N$ and the problem is stated as

$$\begin{aligned} u_1 &= 1 \\ u_i - u_{i-1} &= 0, \;\; i = 2, \dots, N. \end{aligned} \tag{5}$$

The exact solution to this problem is a uniform field $u_i = 1$. ODIL reformulates this problem as minimization of the following loss function

$$\mathcal{L}(u) = \sum_{i=2}^{N} (u_i - u_{i-1})^2 + (u_1 - 1)^2, \tag{6}$$

which has the gradient

$$\begin{aligned} \frac{1}{2}\frac{\partial \mathcal{L}}{\partial u_j} &= \sum_{i=2}^{N} (u_i - u_{i-1})(\delta_{i,j} - \delta_{i-1,j}) + (u_1 - 1)\delta_{1,j} \\ &= \begin{cases} -u_2 + 2u_1 - 1, & j = 1 \\ -u_{j-1} + 2u_j - u_{j+1}, & j = 2, \dots, N-1 \\ u_N - u_{N-1}, & j = N \end{cases} \end{aligned} \tag{7}$$

where $\delta_{i,j} = 1$ if $i = j$ and 0 otherwise. The gradient $\frac{\partial \mathcal{L}}{\partial u_i}$ at each point $i$ only depends on the values in its immediate neighbors $u_{i-1}$, $u_i$, $u_{i+1}$. Therefore, one iteration of the gradient descent method

$$u_i^{s+1} = u_i^s - \eta \frac{\partial \mathcal{L}}{\partial u_i} \tag{8}$$

can propagate the information from the boundary $u_1 = 1$ by at most one point. On the other hand, one iteration of Newton's method solves a linear system

$$\sum_{j=1}^{N} \frac{\partial^2 \mathcal{L}}{\partial u_i \partial u_j}(u_j^{s+1} - u_j^s) = \frac{\partial \mathcal{L}}{\partial u_i}, \quad i = 1, \dots, N \tag{9}$$

and, since the original problem is linear, finds the exact solution from any initial guess. Fig. S2 shows the solution for $N = 5$ at various iterations and convergence history of the gradient descent started from a zero initial guess.

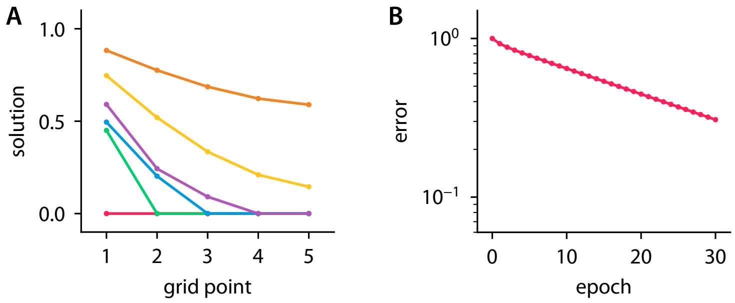


**Fig. S2.** Gradient descent applied to the one-dimensional finite difference equation on a grid of $N = 5$ points. A) Solution at epochs 0 ——, 1 ——, 2 ——, 3 ——, 10 ——, and 30 ——. B) History of the $L_2$ error.

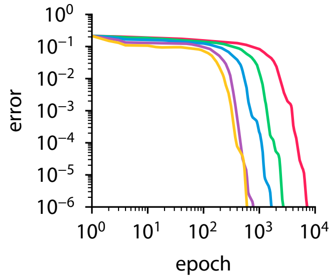


**Fig. S3.** Multigrid decomposition for the lid-driven cavity flow. Computation at Re = 100 on a grid of 65 × 65 cells. Convergence history of L-BFGS-B depending on the number of levels in the multigrid hierarchy showing the $L_2$ error in velocity $u$ relative to the solution of the discrete problem. The number of iterations required to reach an error of $10^{-3}$ reduces from 3840 with one level (65 cells) to 320 with five levels (65, 33, 17, 9, and 5 cells).

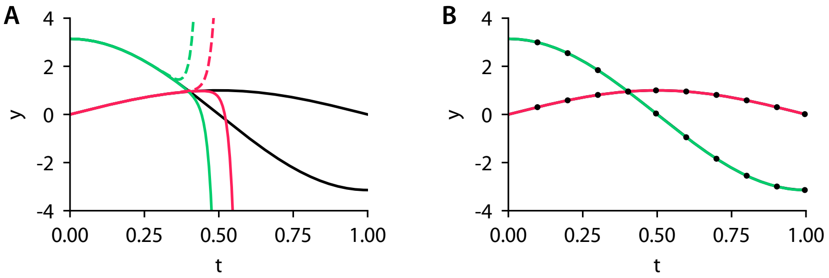


**Fig. S4.** Notorious test problem. Parameter inference from an unstable solution (J. T. Betts, *Practical methods for optimal control and estimation using nonlinear programming*. SIAM, 2010.) of $\dot{y}_1 = y_2$, $\dot{y}_2 = \mu^2 y_1 - (\mu^2 + p^2)\sin pt$ with $\mu = 60$ and initial conditions $y_1 = 0$ and $y_2 = \pi$. A) Solution $y_1$ ——— and $y_2$ ——— of the forward problem with $p = \pi$ obtained using explicit Runge-Kutta. Solution $y_1$ - - - - and $y_2$ - - - - with perturbed initial conditions $y_1 = 0$, $y_2 = \pi + 10^{-10}$. Reference solution $y_1 = \sin \pi t$, $y_2 = \pi \cos \pi t$ ———. B) Solution $y_1$ ——— and $y_2$ ——— of the inverse problem with reference solution imposed in 10 points • obtained using ODIL optimized with L-BFGS with 5000 iterations on a grid of 128 cells. The inferred value of $p$ has an error of $|p - \pi| = 0.00022$.

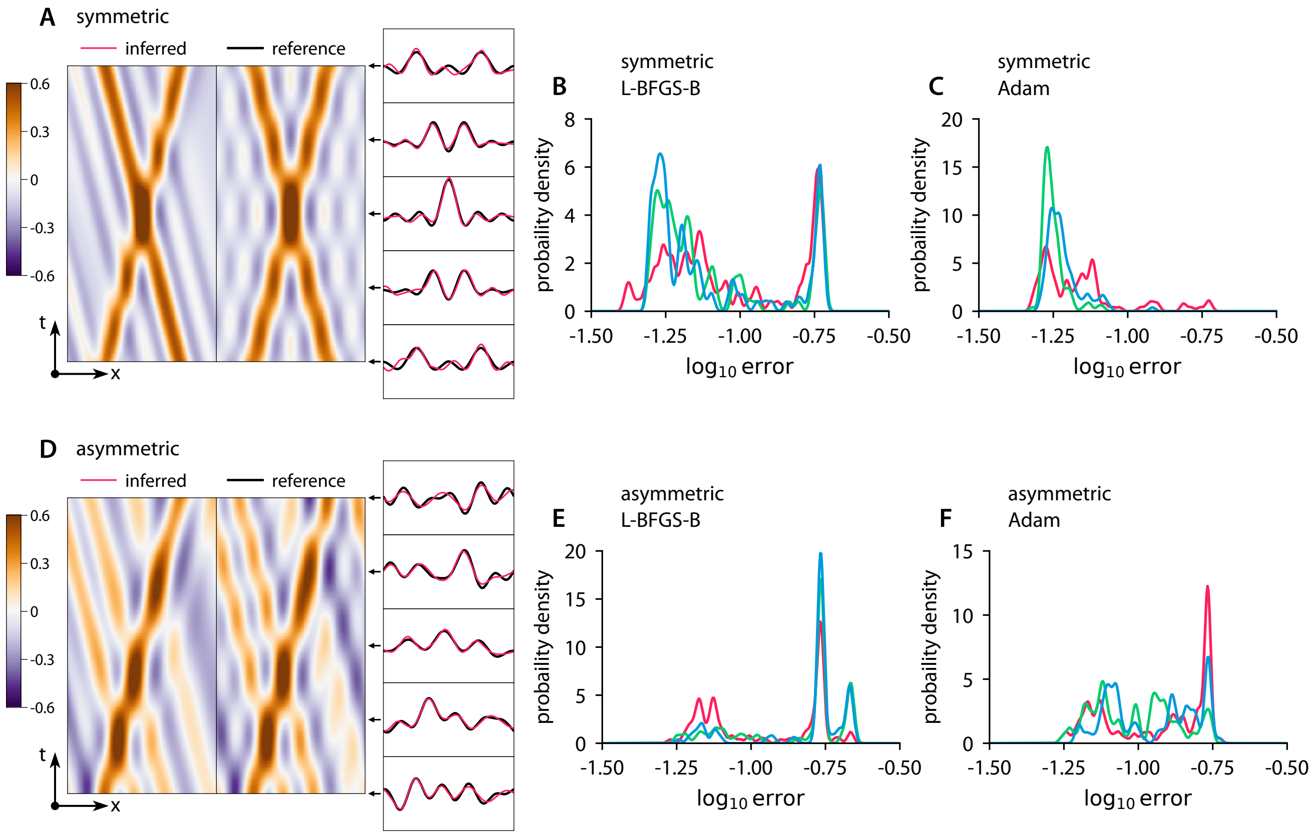


**Fig. S5.** Effect of normalization and initialization in PINN. Wave equation with symmetric solution $u(x,t) = \frac{1}{10}\sum_{k=1}^{5}(\cos(x-t+0.5)\pi k + \cos(x+t+0.5)\pi k)$ A, B, C) and asymmetric solution $u(x,t) = \frac{1}{10}\sum_{k=1}^{5}(\cos(x-t+0.5)\pi k + \cos(x+t+0.5+2k)\pi k)$ D, E, F). Baseline —— without normalization and initialization by $U[-\sqrt{1/d_i}, \sqrt{1/d_i}]$. Normalization —— of inputs to zero mean and unit variance by transformation $t \leftarrow (2t-1)\sqrt{3}$, $x \leftarrow x\sqrt{3}$. Normalization and initialization by Glorot —— $U[-\sqrt{3/d_i}, \sqrt{3/d_i}]$. The problem is solved using a neural network of size $25 \times 25$. For the normalization of the weights, $d_i$ denotes the number of input elements in each layer. Adam with a learning rate of 0.005 and L-BFGS-B with 50 terms in the limited memory matrix. Showing the probability density of the error achieved after 1000 epochs estimated from 100 samples of the random seed.

Observations:

(a) Normalization may worsen or improve the performance depending on the case, but the overall effect is comparable to that of changing the random seed.

(b) Changing the initialization $U[-\sqrt{1/d_i}, \sqrt{1/d_i}]$ to $U[-\sqrt{3/d_i}, \sqrt{3/d_i}]$ has no significant effect.

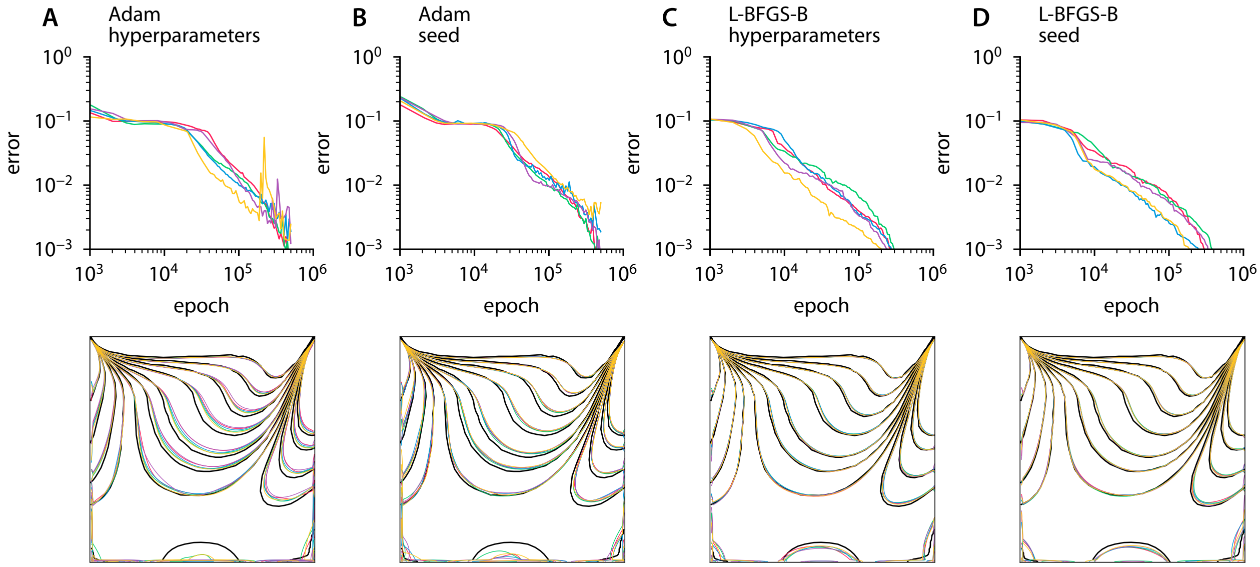


**Fig. S6.** Effect of hyperparameters of PINN. Training history and vorticity contours. A, C) Initialization and architecture. Glorot uniform $32 \times 32 \times 32$ ——, He uniform $32 \times 32 \times 32$ ——, LeCun uniform $32 \times 32 \times 32$ ——, Glorot uniform $32 \times 32 \times 32 \times 32$ ——, and Glorot uniform $32 \times 32 \times 32 \times 32 \times 32$ ——. B, D) Random seed 100 ——, 110 ——, 120 ——, 130 ——, and 140 —— with He uniform $32 \times 32 \times 32$. A, B) Adam with a learning rate of 0.001. C, D) L-BFGS-B with 10 terms in the limited memory matrix.

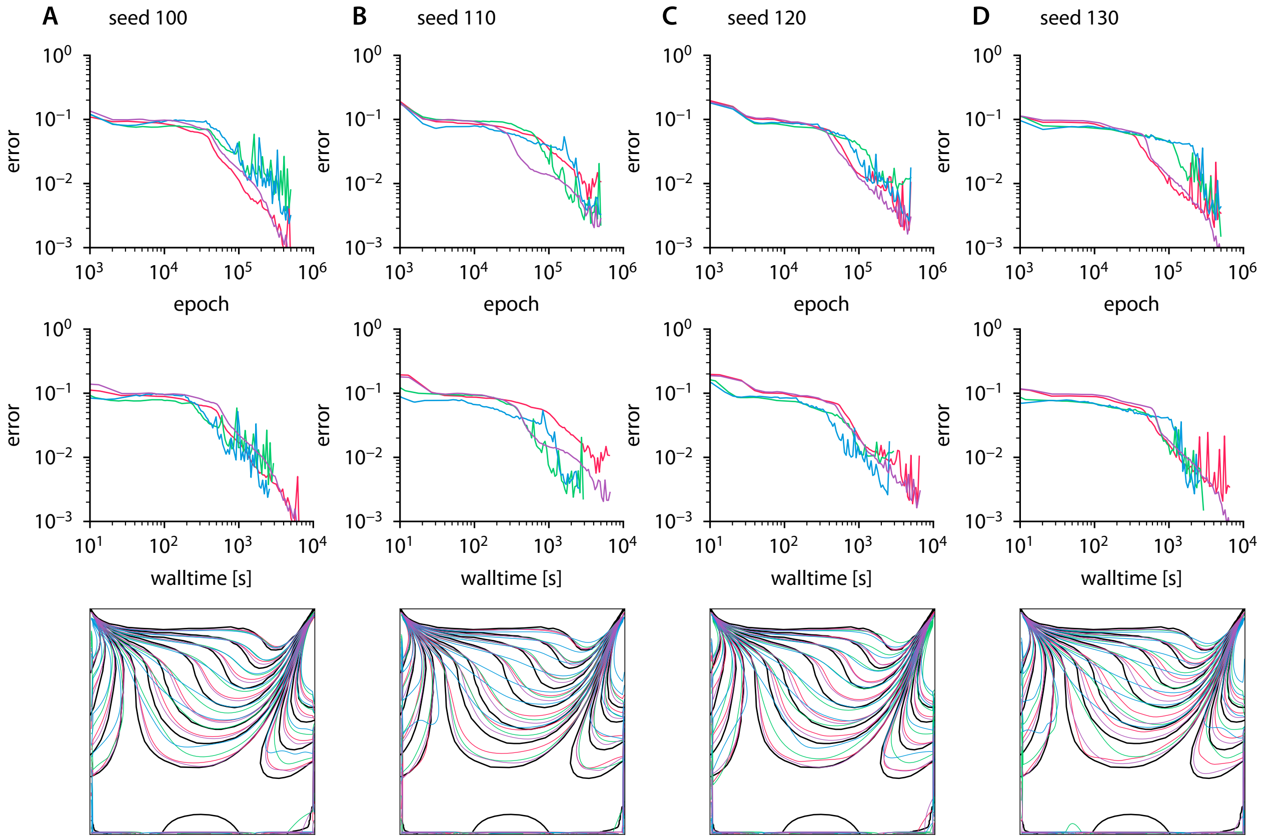


**Fig. S7.** Effect of resampling of collocation points in PINN (mini-batch training). 10000 (red line), 1000 (green line), and 100 (blue line) collocation points inside the domain resampled every 100 epochs; 10000 (purple line) fixed points inside the domain without resampling. Each column corresponds to one value of the random seed and shows the error in $x$-velocity versus epoch, error in $x$-velocity versus walltime, and vorticity contours. Optimization using Adam with a learning rate of 0.001 on a GPU. Observations:

(a) Resampling with fewer points (mini-batch training) slows down the convergence and increases the variance of the error.

(b) The gained reduction of the per-iteration cost is not sufficient to justify the use of resampling.

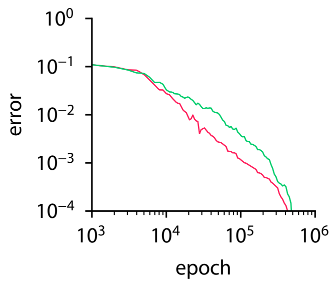


**Fig. S8.** Effect of parameters of L-BFGS-B for PINN. Training history with 10 —— and 50 —— terms in the limited memory matrix of L-BFGS-B. Showing the error in the $x$-velocity relative to the field obtained at the last epoch 500000. Using 10 terms results in faster convergence.

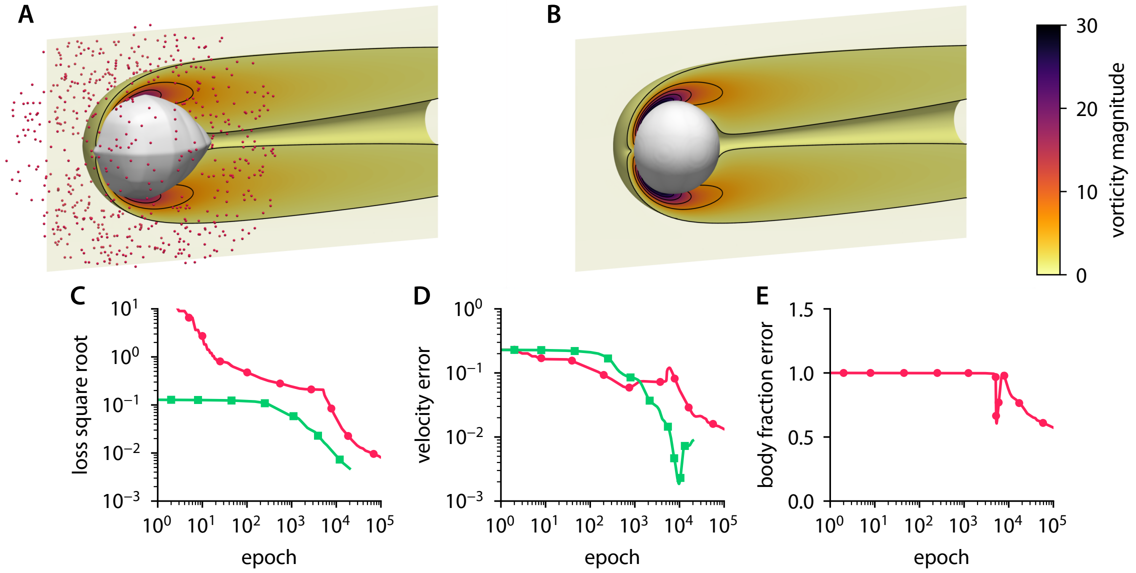


**Fig. S9.** Inferring body shape from velocity measurements for a flow past a sphere using ODIL on a grid of $257 \times 129 \times 129$ cells. Inferred (A) and reference (B) body shape and contours of vorticity magnitude. The velocity measurements are imposed in 684 points (red dots) chosen at a distance from the body. History of the square root of the loss (C), root-mean-square error in $x$-velocity (D), and body fraction $\chi$ normalized by the mean of reference (E) for the inverse problem ─●─ and forward problem ─■─. Optimization using L-BFGS with 10 terms in the limited memory matrix. The error is relative to the solution of the forward problem after 10000 epochs.

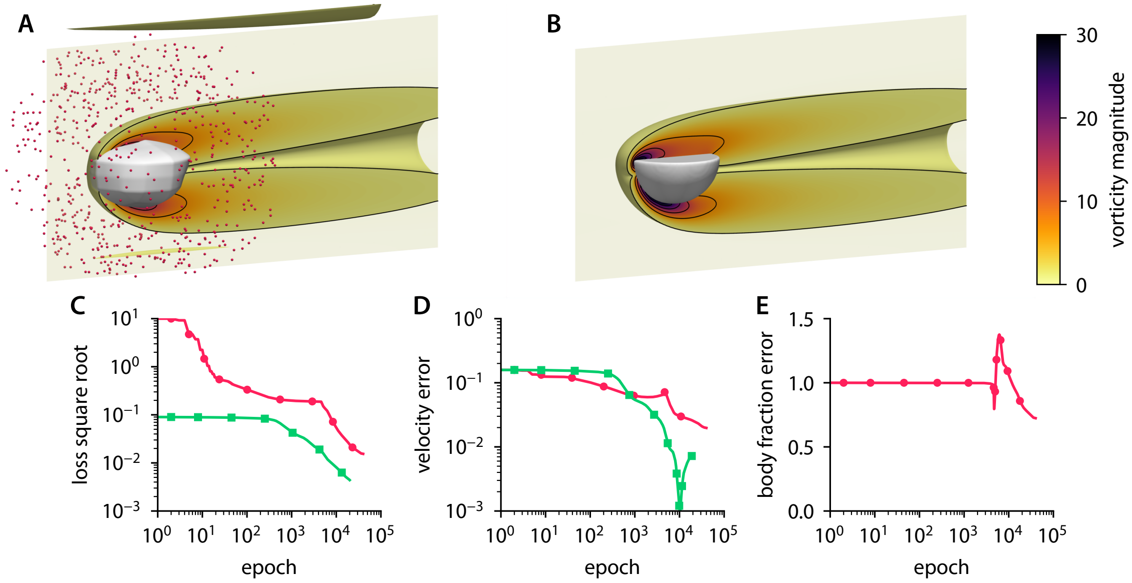


**Fig. S10.** Inferring body shape from velocity measurements for a flow past a hemisphere using ODIL on a grid of $257 \times 129 \times 129$ cells. Inferred (A) and reference (B) body shape and contours of vorticity magnitude. The velocity measurements are imposed in 684 points (red dots) chosen at a distance from the body. History of the square root of the loss (C), root-mean-square error in $x$-velocity (D), and body fraction $\chi$ normalized by the mean of reference (E) for the inverse problem (red line, circle markers) and forward problem (green line, square markers). Optimization using L-BFGS with 10 terms in the limited memory matrix. The error is relative to the solution of the forward problem after 10000 epochs.

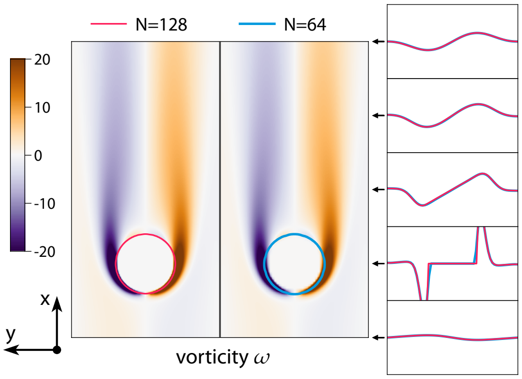


**Fig. S11.** Flow past a circle solved using ODIL on a grid of $2N \times N$ cells for $N = 64$ and 128.

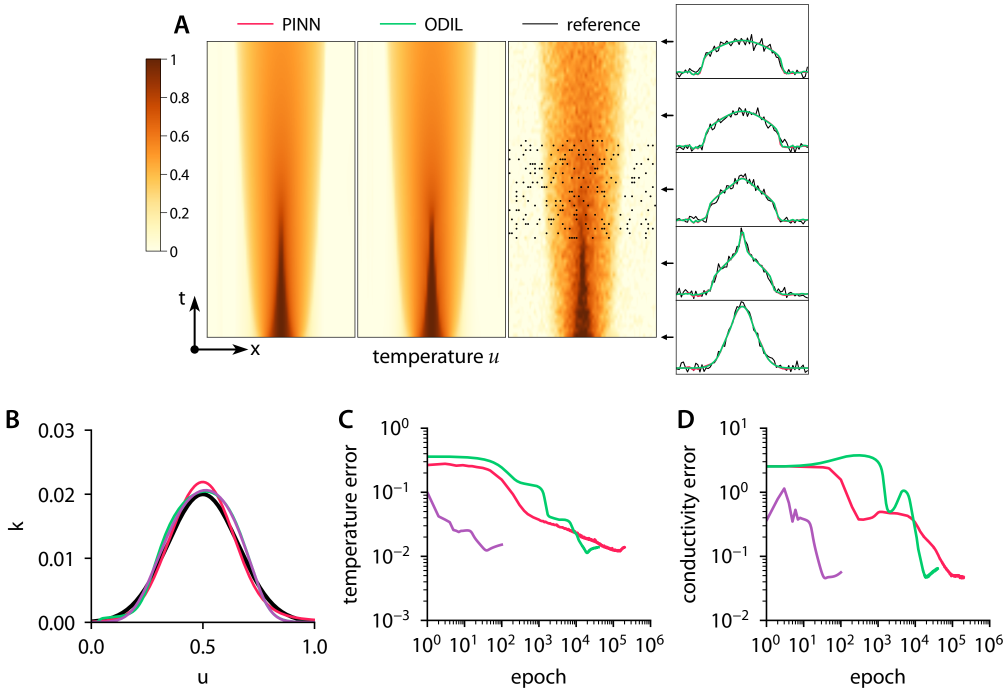


**Fig. S12.** Inferring conductivity from noisy temperature measurements using ODIL and PINN. Temperature field (A) and conductivity function (B) inferred by PINN with Adam ——, ODIL with Adam ——, and ODIL with Newton —— compared to reference ——. The reference solution is from forward problem solved using ODIL on a finer grid. The temperature measurements in 200 points (black dots) are taken from the reference solution perturbed by Gaussian noise with $\sigma = 0.05$. History of root-mean-square error in temperature (C) and conductivity (D) relative to unperturbed reference solution.

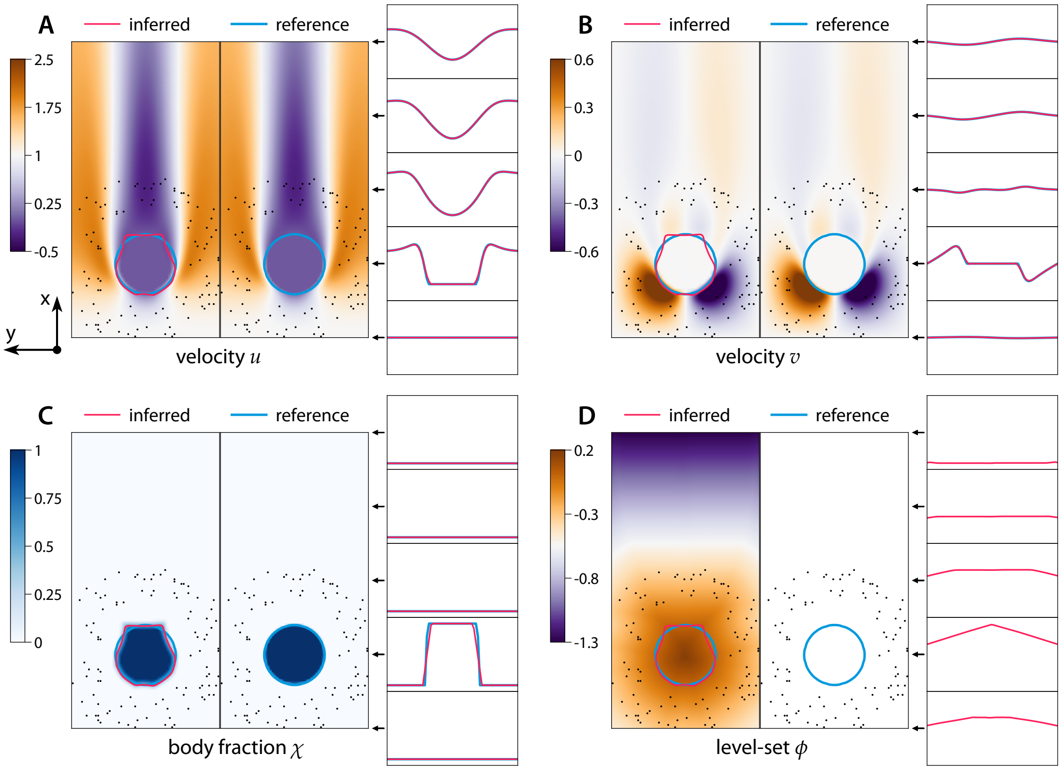


**Fig. S13.** Inferring body shape from velocity measurements for a flow past a circle at Re = 60 using ODIL. Inferred ——— and reference ——— velocity $u$ (A), velocity $v$ (B), body fraction $\chi$ (C), and level-set function $\phi$ (D) overlapped by contours of body fraction $\chi = 0.5$.

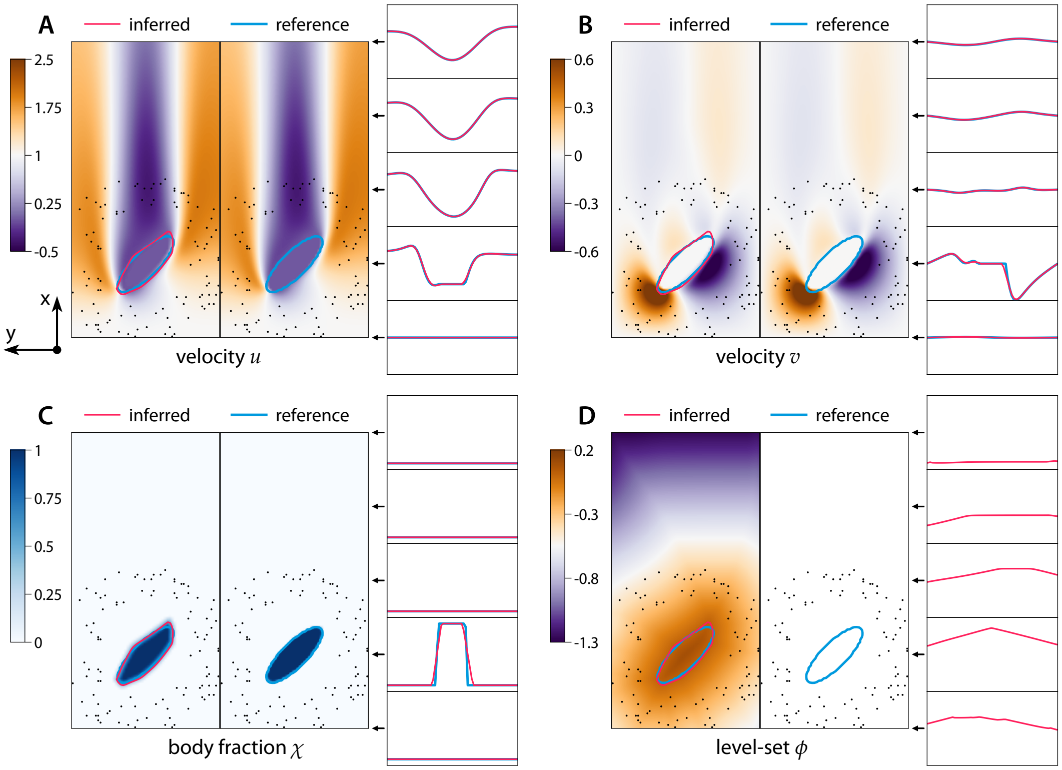


**Fig. S14.** Inferring body shape from velocity measurements for a flow past an ellipse at Re = 60 using ODIL. Inferred ── and reference ── velocity $u$ (A), velocity $v$ (B), body fraction $\chi$ (C), and level-set function $\phi$ (D) overlapped by contours of body fraction $\chi = 0.5$.

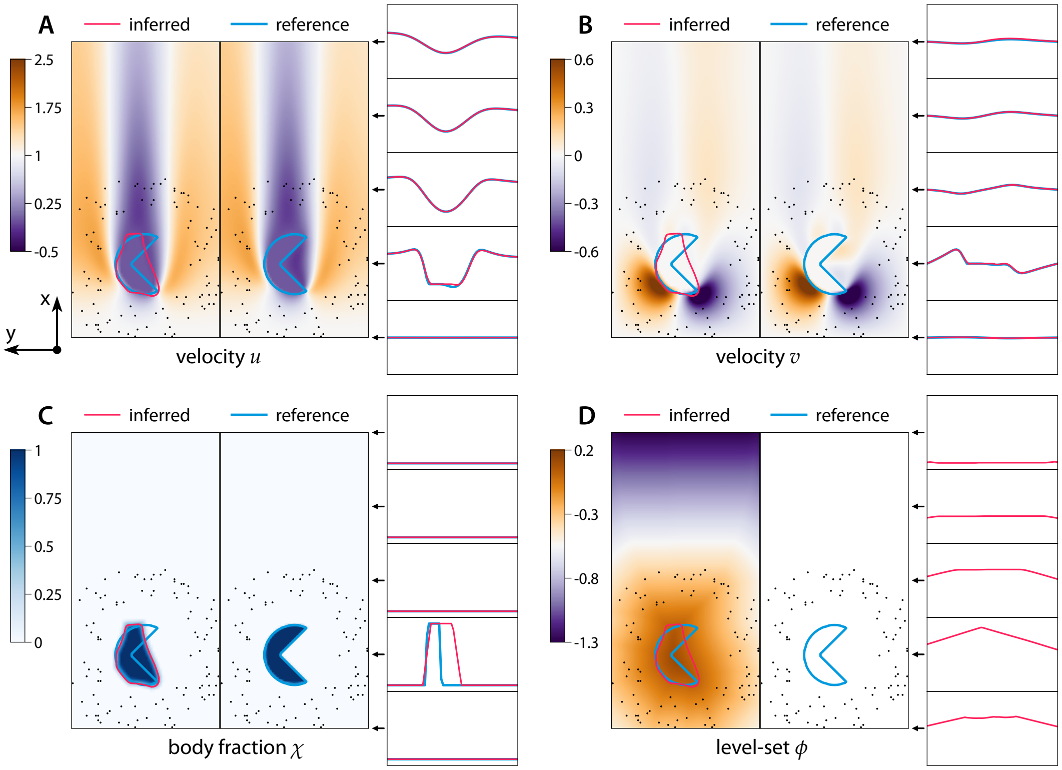


**Fig. S15.** Inferring body shape from velocity measurements for a flow past a non-convex body at Re = 60 using ODIL. Inferred ─── and reference ─── velocity $u$ (A), velocity $v$ (B), body fraction $\chi$ (C), and level-set function $\phi$ (D) overlapped by contours of body fraction $\chi = 0.5$.
